# A probabilistic computational framework for the prediction of corrosion-induced cracking in large structures

**DOI:** 10.1038/s41598-022-25477-8

**Published:** 2022-12-03

**Authors:** Guofeng Qian, Karnpiwat Tantratian, Lei Chen, Zhen Hu, Michael D. Todd

**Affiliations:** 1 Department of Structural Engineering, University of California, San Diego, CA 92093-0085 USA; 2 Department of Mechanical Engineering, University of Michigan-Dearborn, Dearborn, MI 48128-1491 USA; 3 Department of Industrial and Manufacturing Systems Engineering, University of Michigan-Dearborn, Dearborn, MI 48128 USA

**Keywords:** Civil engineering, Structural materials, Computational methods

## Abstract

Corrosion can initiate cracking that leads to structural integrity reduction. Quantitative corrosion assessment is challenging, and the modeling of corrosion-induced crack initiation is essential for model-based corrosion reliability analysis of various structures. This paper proposes a probabilistic computational analysis framework for corrosion-to-crack transitions by integrating a phase-field model with machine learning and uncertainty quantification. An electro-chemo-mechanical phase-field model is modified to predict pitting corrosion evolution, in which stress is properly coupled into the electrode chemical potential. A crack initiation criterion based on morphology is proposed to quantify the pit-to-cracking transition. A spatiotemporal surrogate modeling method is developed to facilitate this, consisting of a Convolution Neural Network (CNN) to map corrosion morphology to latent spaces, and a Gaussian Process regression model with a nonlinear autoregressive exogenous model (NARX) architecture for prediction of corrosion dynamics in the latent space over time. It enables the real-time prediction of corrosion morphology and crack initiation behaviors (whether, when, and where the corrosion damage triggers the crack initiation), and thus makes it possible for probabilistic analysis, with uncertainty quantified. Examples at various stress and corrosion conditions are presented to demonstrate the proposed computational framework.

## Introduction

Stress corrosion and stress corrosion cracking (SCC) damage have been significant types of deterioration in large civil infrastructure^1,2^. Over decades of service, components are exposed to variable service loads and a changing natural environment at the same time. Such operational and environmental loading has significant influence on the evolution of local pitting corrosion^3^ and SCC initiation, which may ultimately lead to structural failure. Furthermore, because of the inevitable variability of mechanical and electrochemical behavior affected by the environment, pitting corrosion and corrosion-induced crack initiation are almost implausible to compute accurately in a deterministic sense. Instead, probabilistic analysis combined with degradation model provides reasonable estimates of the damage growth and prediction of the life cycle of the structures in service^4^. However, because of the intrinsic complexity of the multi-physics corrosion simulation (usually involves multiple coupled partial differential equations and takes hours to solve^3^), it is computational prohibitive at present to perform probabilistic studies which requires thousands of simulations. Therefore, a probabilistic study framework with reasonable computation cost is needed to comprise digital twins tasked for risk-informed lifecycle management subject to corrosion damage.

The phase-field method is a powerful mesoscale method to analyze the spatiotemporal evolution of microstructure. This method can be extended by coupling with an electrochemical reaction model to simulate the phase evolution in the corrosion process. The phase change is described by a continuous variable such that an explicit treatment of the interface may be avoided. LQ Chen proposed a phase-field for microstructure evolution^5^. Nonlinearity is introduced to this model by considering the chemical reaction kinetics^6^. Multiple applications of the phase-field method on corrosion have been proposed and studied^3,7–12^. Mai et al.^3^ proposed a phase-field model to simulate pitting corrosion. Local free energy was described with the Kim-Kim-Suzuki (KKS) model^13^. However, the electrical potential distribution for ion concentration was not considered. Ansari et al.^14^ took the influence of insoluble corrosion products into consideration in phase field corrosion modeling. Chen et al.^15^ coupled mechanical influence to the electrochemical system by multiplying an interpolation function of the order parameter with the original mechanical energy. The mechanical term in the electrochemical potential that was calculated similarly by taking variation of total free energy was a square term of the elastic strain tensor. Chen et al.^8^ also involved mechanical effect coupled with galvanic influence. The change of chemical potential of electrode due to mechanical deformation is considered as the sum of mechanical energy density and mechanical potential. All current attempts for coupling mechanical influence into phase field corrosion modeling led to a similar behavior under tensile or compressive stress. Nevertheless, multiple experiments showed obvious differences between the corrosion under tensile and compressive stress^16–18^. Such differences between tensile stress and compressive stress is rarely studied or discussed in the phase-field modeling area, and it will be one aspect considered in this work.

Stress corrosion cracking (SCC) was also simulated with phase-field method. Mai et al.^19^ modified the kinetics parameter term by assuming a linear relationship with the SCC growth velocity. Nguyen et al.^9^ took ion concentration and elastic energy densities into consideration in total free energy. However, the transition from corrosion to crack and the relationship between crack initiation and applied mechanical load has not been studied yet. More crucially, multiple experimental data show that the uncertainties at the crack initiation stage is obviously higher than during the stable propagation stage^12,13^. A probabilistic analysis framework which can take the uncertainty sources at the crack initiation stage into consideration is thus an important gap to fill, and this is also addressed in this work.

Existing high-fidelity phase-field models for corrosion problems are inherently computationally expensive because they need to solve a system of coupled partial differential equations. To predict corrosion evolution for large, complex structures, more computationally efficient modeling is needed. To relieve this burden, a lot of work has been done on leveraging computing architectures^20,21^ and numerical strategies^22^. Different kinds of surrogate models have been built to accelerate the prediction of phase evolution including Green’s function solution^23^ or Bayesian optimization^24^. However, the balance between the accuracy and the computational efficiency is still a challenge for these methods. Green’s function solution is computationally efficient but the accuracy for complex models is not satisfactory, while Bayesian optimization can provide more accurate results at a substantially higher computational cost. Machine learning based methods have been proposed recently to build a surrogate model for microstructure evolution^25^. Machine learning techniques were implemented to predict the phase evolution after dimensionality reduction. These models performed well in predicting the statistical information like microstructure’s autocorrelation. Yet, the direct phase evolution prediction results are not accurate. The interface between two phases in the evolution is not clear due to the error in prediction^25^, which can be problematic because the interface is of great interest in the corrosion process and is crucial for crack initiation prediction.

This work overcomes the limitations of current pitting corrosion simulation methods by making the following contributions. To accelerate the physics-based corrosion propagation simulation for probabilistic study, a surrogate model combining Convolutional neural network (CNN) and Gaussian process (GP)-based nonlinear autoregressive network with exogenous inputs (NARX) is proposed to predict the corrosion growth and potential crack initiation time and location. This model can provide accurate prediction with negligible computing time, enabling probabilistic methods for risk-informed lifecycle management. An electro-chemo-mechanical phase-field model coupled with mechanical stress is also developed based on the generalized chemical potential. This model can capture the influence of different kinds of mechanical loading (tensile, compressive, and shear stress) on pitting corrosion growth, by embedding the stress term into the overpotential in the Butler-Volmer equation. A SCC initiation criterion based on a Von-mises limit state is proposed and compared with the other SCC initiation criteria^26^ based on the Tsujikawa–Kondo condition^27^.

**Figure 1:**
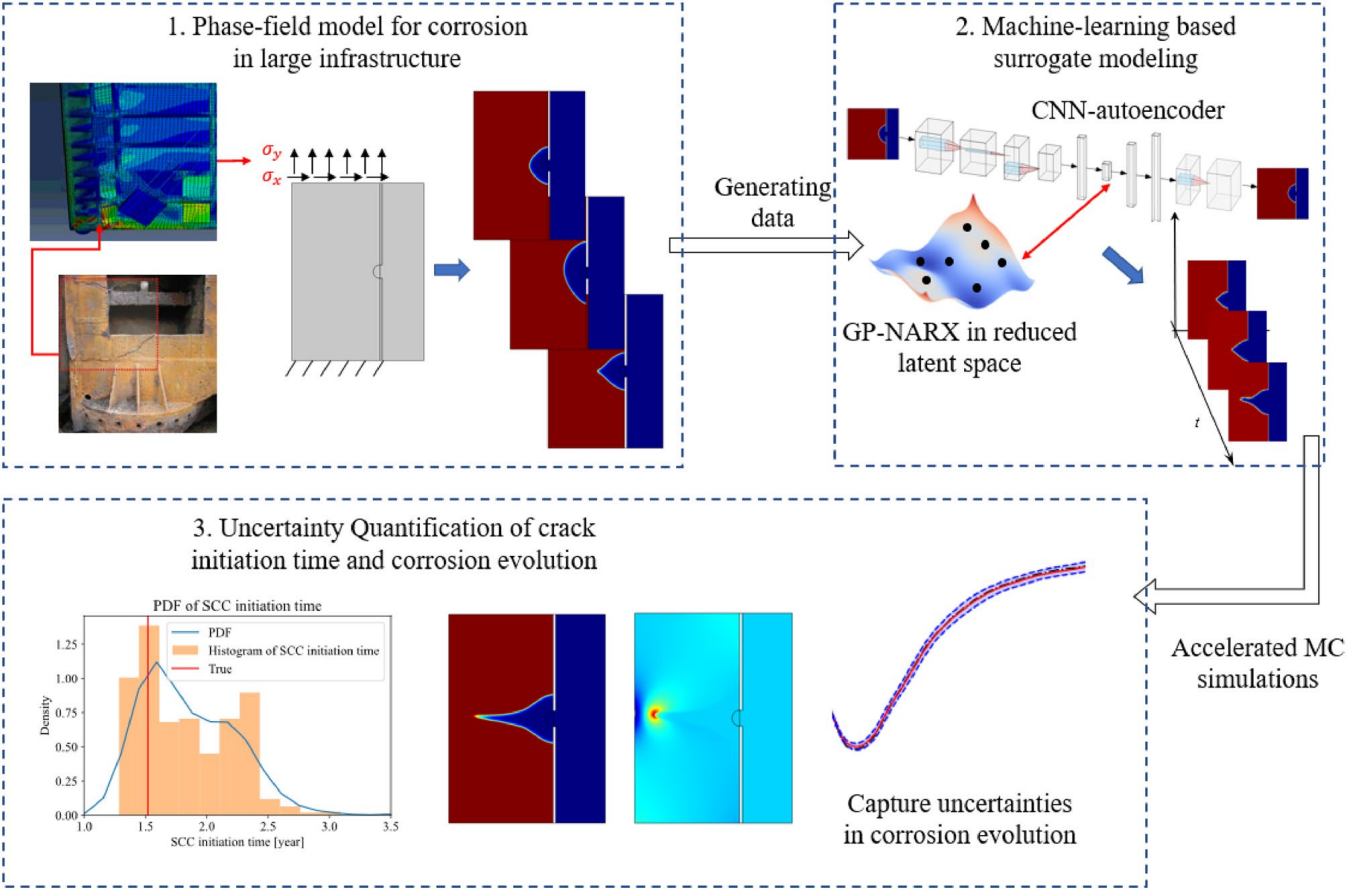
Overview of the proposed surrogate model-accelerated probabilistic analysis framework for corrosion-to-cracking prediction in large structures.

Figure 1 gives an overview of the proposed corrosion-to-cracking simulation framework. As shown in Step 1 of Fig. 1, a calibrated high-fidelity phase-field simulation model is used to generate corrosion morphology images for different training samples of external loads, reaction constant, and diffusion coefficient. These four parameters—mechanical normal stress, mechanical shear stress, reaction constant, and diffusion coefficient—in addition to the initial morphology, represent the different environmental conditions for the structure in service.

The results of the simulation are corrosion morphology images over time. Then, the high-dimensional corrosion morphology images are mapped into low-dimensional data in latent space with a convolutional neural network (CNN) autoencoder (see Step 2 in Fig. 1). Next, a Gaussian process regression model with a nonlinear autoregressive model with exogenous inputs, named GP-NARX, is utilized to learn the corrosion evolution dynamics in the latent space. GP-NARX is chosen because it can capture the uncertainty of the surrogate model prediction in a closed form, facilitating uncertainty quantification of the overall surrogate model prediction performance later. After that, the low-dimensional prediction results in the latent space are decompressed to the original high-dimensional corrosion morphology images with decoder trained together with autoencoder. Afterwards, the surrogate model is used to complete Monte-Carlo simulations to quantify the uncertainty in the crack initiation time caused by the surrogate model uncertainty as Fig. 1 shows in Step 3. Such uncertainties in the latent space originally are propagated into the morphology space. Proposed SCC initiation criterion is then implemented on the Monte-Carlo simulation results to get the probability distribution function of the crack initiation time. With the highly efficient simulation capability, the proposed framework can be extended to incorporate many other uncertainty sources in the future, such as uncertainty in crack initiation stage, uncertainty in the load condition, etc.

The remainder of this paper is organized as follows: Section “Pitting corrosion modeling and pit-to-crack transition” presents the developed pitting corrosion simulation using a phase-field model, and pit-to-crack transition analysis based on pitting corrosion simulation; Section “Surrogate model of pitting corrosion simulation and crack initiation” describes the proposed surrogate modeling method for pitting corrosion simulation. Section “Results and discussions” gives the results of pitting corrosion simulation, pit-to-crack transition analysis, and probabilistic crack initiation time and location analysis. Finally, Section “Conclusions” concludes and discusses the presented work.

## Pitting corrosion modeling and pit-to-crack transition

### Stress corrosion and SCC mechanism

**Figure 2:**
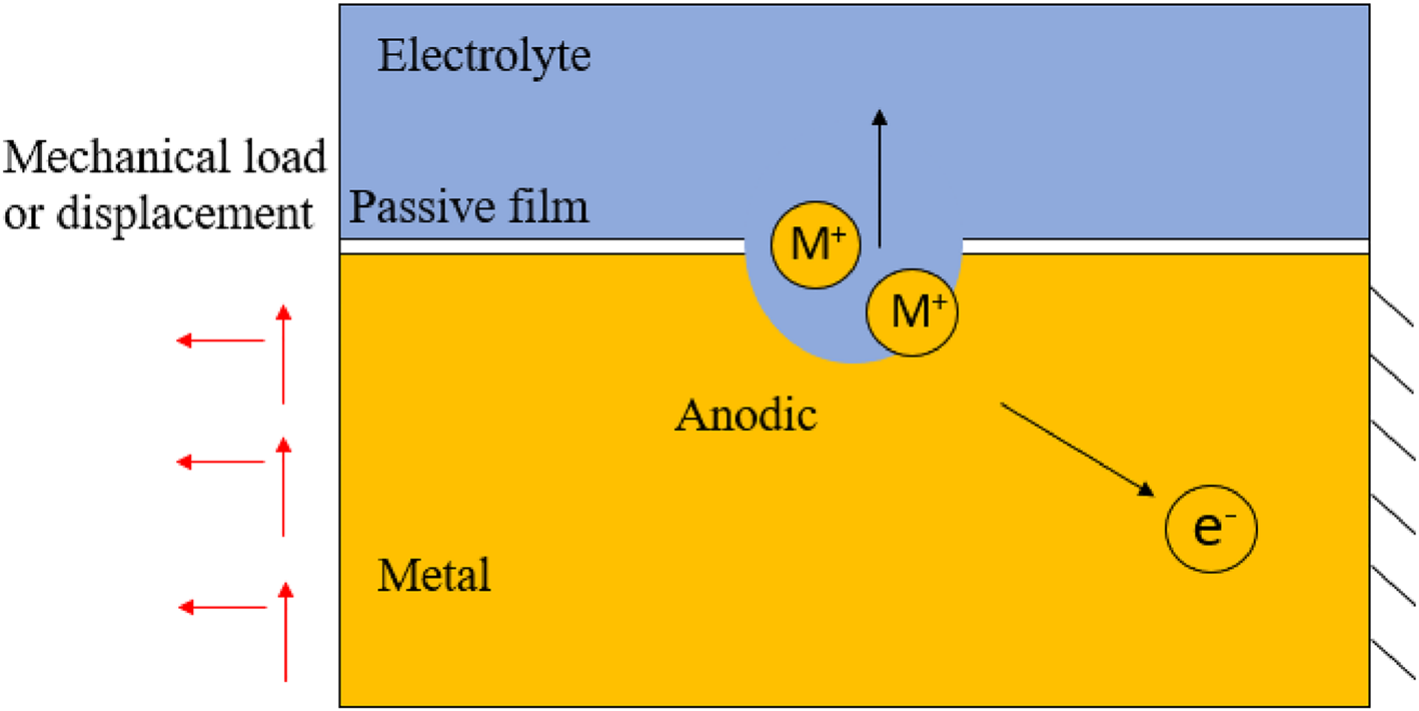
Schematics of stress corrosion.

The stress corrosion process, as shown in Fig. 2, starts from the local breakage of the passive film. Under a corrosive environment, the metal is corroded and produce cations (${M}^{+}$) into the electrolyte as well as the electrons in the electrode. During service life, complex mechanical loading conditions typically occur in different locations. However, mechanical loading changes the chemical potential of the electrode (metal), affecting the corrosion process. Stress concentration at the tip of the pitting corrosion amplifies the influence from the mechanical stress. In order to properly quantify the contribution from mechanical load, a generalized potential is introduced.

### Generalized chemical potential and kinetics in corrosion system with mechanical stress

The generalized chemical potential in the electrochemical system is expressed as

1 $$\mu = {\mu }_{t}+{\mu }_{el}+{\mu }_{gr}+{\mu }_{me},$$

where $\mu$ is the well-defined chemical potential, $\mu$ usually consists of thermal potential ${\mu }_{t}$, electrical potential ${\mu }_{el}$, gradient energy density or gradient potential ${\mu }_{gr}$, and mechanical energy density or mechanical potential ${\mu }_{me}$.

The influence of mechanical deformation on corrosion process originates from the potential change due to mechanical deformation on the surface of the solid^28,29^. According to Gibbs–Duhem equation^30^, the differential of chemical potential dependence is described as

2 $$\sum_{i}{N}_{i}d{\mu }_{i}=-SdT+VdP$$

where $N$ is molarity, $V$ is the volume, $S$ is the entropy, $T$ is the temperature and $P$ is the pressure, calculated with hydrostatic stress by $P=-\frac{1}{3}\sum_{k=1}^{3}{\sigma }_{kk}$.

Assuming the metal response is linearly elastic^31^, the linear expression for ${\mu }_{me}$ by integrating Eq. (2) follows expression in mechanochemistry area by Gutman^28,29^

3 $$\mu _{{me}} = \int\limits_{{P_{1} }}^{{P_{2} }} {V(P)} dP \approx \Delta PV_{m} ,$$

where ${P}_{1},{P}_{2}$ are the initial and end pressure and ${V}_{m}$ is the molar volume.

The thermal potential is expressed as

4 $${\mu }_{t}=g\left(\overline{c }\right)+{c}_{0}RT\left({\overline{c} }_{+}\mathrm{ln}{\overline{c} }_{+}+{\overline{c} }_{-}\mathrm{ln}{\overline{c} }_{-}\right)+\sum_{i}{c}_{i}{\mu }_{i}^{\Theta },$$

where ${\varvec{c}}=\left\{c, {c}_{+},{c}_{-}\right\}$ is a set of concentrations for the metal atom, metal cations, and electron, respectively. Further, $\overline{{\varvec{c}} }$ is defined as the set of dimensionless concentrations as $\left\{\overline{c }=\frac{c}{{c}_{s}},{\overline{c} }_{+}=\frac{{c}_{+}}{{c}_{0}},{\overline{c} }_{-}=\frac{{c}_{-}}{{c}_{0}}\right\}$, where ${c}_{s}$ is the site density of the metal iron and ${c}_{0}$ is the bulk concentration of electrolyte solution, $R$ is molar gas constant, and $T$ is the temperature. The double-well function $g\left(\overline{c }\right)=W{\overline{c} }^{2}{\left(1-\overline{c }\right)}^{2}$ is used to describe the transition between electrode $\left(\overline{c }=1\right)$ and electrolyte $\left(\overline{c }=0\right)$. $W$ represents barrier height of corrosion. ${\mu }_{i}^{\Theta }$ is the reference chemical potential of spices $i$.

The electric potential ${\mu }_{el}$ is expressed as

5 $${\mu }_{el}={\rho }_{e}\phi ,$$

where $\phi$ is the electrostatic potential, ${\rho }_{e}$ is the charge density which can be expressed as ${\rho }_{e}=F{z}_{i}{c}_{i}$ where $F$ is Faraday’s constant, ${z}_{i}$ is the valence and ${c}_{i}$ is the concentration of species $i$.

The interfacial potential ${\mu }_{gr}$ is given by taking partial derivative of the interface energy as^6^

6 $${\mu }_{gr}={g}^{^{\prime}}\left(\overline{c }\right)-\kappa {\nabla }^{2}\overline{c },$$

where $\kappa$ is interface coefficient.

According to previous formulation of electrochemical reaction kinetics, the reaction rate, ${R}_{e}$ of corrosion is expressed as the difference between forward $\left({S}_{1}\to {S}_{2}\right)$ and backward $\left({S}_{2}\to {S}_{1}\right)$ reactions in a form of Butler–Volmer equation as

7 $${R}_{e}={k}_{0}\left(\mathrm{exp}\left[\frac{-\left({\mu }_{t}^{ex}-{\mu }_{1}\right)}{RT}\right]-\mathrm{exp}\left[\frac{-\left({\mu }_{t}^{ex}-{\mu }_{2}\right)}{RT}\right]\right),$$

where ${\mu }_{1}$ and ${\mu }_{2}$ refers to the total chemical potential at state 1 and state 2 respectively, ${\mu }_{t}^{ex}$ is the activation barrier, ${k}_{0}$ is the reaction constant.

Based on the potentials Eqs. (1–6) we defined previously, we can get the potential expressions of initial state 1 (electrode) and later state 2 (electrolyte) by different components in the corrosion reaction, $M\to {M}^{n+}+n{e}^{-}$

8a $${{\mu }_{1}=\mu }_{M}={\mu }_{M}^{t}+{\mu }_{M}^{me}+{\mu }_{M}^{gr}={\mu }_{gr}+{\mu }_{M}^{\Theta }+{\mu }_{me},$$

8b $${\mu }_{2}={\mu }_{{M}^{n+}}^{t}+{n\mu }_{el}=RT\mathrm{ln}{a}_{{M}^{n+}}+{\mu }_{{M}^{n+}}^{\Theta }+nF{\phi }_{s}+nRT\mathrm{ln}{a}_{e}+{n\mu }_{e}^{\Theta }-nF{\phi }_{e},$$

where ${\phi }_{s}$ and ${\phi }_{e}$ are the electrostatic potential in the solution and the electrode respectively, ${a}_{M}$, ${a}_{{M}^{n+}}$ and ${a}_{e}$ are the activities of the components. The activity for electrons is unity assuming that the electrolyte solution is dilute. The interfacial potential difference is $\Delta \phi ={\phi }_{e}-{\phi }_{s}$. At the equilibrium, the potential difference $\Delta \mu ={\mu }_{2}-{\mu }_{1}=0$ according to the Nernst equation

9 $$\Delta {\phi }^{eq}=\frac{{\mu }_{{M}^{n+}}^{\Theta }+{n\mu }_{e}^{\Theta }-{\mu }_{M}^{\Theta }+RT\mathrm{ln}{\overline{c} }_{+}+{\mu }_{me}-{\mu }_{gr}}{nF}.$$

Outside equilibrium, the reaction is driven by the overpotential, $\eta$, which is defined as

10 $$\eta =\Delta \phi -\Delta {\phi }^{eq}.$$

Substituting Eq. (10) with Eq. (9), $\eta$ is expressed as

11 $$\eta =\Delta \phi -\frac{{\mu }_{{M}^{n+}}^{\Theta }+{n\mu }_{e}^{\Theta }-{\mu }_{M}^{\Theta }}{nF}-\frac{RT\left(\mathrm{ln}{\overline{c} }_{+}\right){-\mu }_{gr}}{nF}+\frac{{\mu }_{me}}{nF},$$

where the second term represents the standard potential difference between reactants and products, the third term expresses the concentration overpotential, and the third term is the influence of mechanical elastic energy density. The total overpotential can be separated into activation overpotential ${\eta }_{a}=\Delta \phi -\frac{{\mu }_{{M}^{n+}}^{\Theta }+{n\mu }_{e}^{\Theta }-{\mu }_{M}^{\Theta }+{\mu }_{me}}{nF}$, concentration overpotential ${\eta }_{c}=-\frac{RT\left(\mathrm{ln}{\overline{c} }_{+}\right){-\mu }_{gr}}{nF}$.

The excess electrochemical potential in the transition state is defined as^32^

12 $${\mu }_{t}^{ex}=RT\mathrm{ln}{\gamma }_{t}+{\mu }_{me}^{ex}+\left(1-\alpha \right){\mu }_{M}^{\Theta }+\alpha \left({\mu }_{{M}^{n+}}^{\Theta }+{n\mu }_{e}^{\Theta }\right),$$

where ${\gamma }_{t}$ is the activity coefficient at the transition state, ${\mu }_{me}^{ex}$ represents the mechanical potential at the transition state, and $\alpha$ is an approximate constant ranging from zero to one called symmetry factor.

The reaction rate can be expressed by substituting Eqs. (8–12) into Eq. (6)

13 $$r=\frac{{k}_{0}}{{\gamma }_{t}}\mathrm{ exp}\left(-\frac{{\mu }_{me}^{ex}}{RT}\right)\times \left\{\mathrm{exp}\left(\frac{{\mu }_{gr}+\left(1-\alpha \right){\eta }_{a}}{RT}\right)-{\overline{c} }_{+}\mathrm{exp}\left(\frac{-\alpha nF{\eta }_{a}}{RT}\right)\right\}.$$

The influence of the interfacial potential concentration gradient at the interface on the corrosion process is usually small comparing to other components in the total chemical potential^33^. A nonlinear relationship for phase transforming is proposed by Liang et al.^6,34^ as

14 $$r={-L}_{\sigma }\left({g}^{^{\prime}}\left(\overline{c }\right)-\kappa {\nabla }^{2}\overline{c } \right)-{L}_{\eta }\left(\mathrm{exp}\left(\frac{\left(1-\alpha \right){\eta }_{a}}{RT}\right)-{\overline{c} }_{+}\mathrm{exp}\left(\frac{-\alpha nF{\eta }_{a}}{RT}\right)\right),$$

where ${L}_{\sigma }=\frac{{k}_{0}}{RT{{\gamma }_{t}c}_{s}}\mathrm{exp}\left(-\frac{{\mu }_{\mathit{me}}^{\mathit{ex}}}{\mathit{RT}}\right)\mathrm{exp}\left(\frac{\left(1-\alpha \right){\eta }_{a}}{RT}\right)$ represents interfacial mobility and ${L}_{\eta }=\frac{{k}_{0}}{{\gamma }_{t}}\mathrm{exp}\left(-\frac{{\mu }_{{\mu }_{me}}^{ex}}{RT}\right)$ represents a reaction coefficient. In the work, we assume the reaction coefficient is a constant value which will be calibrated in Section “Calibration of the phase-field model”.

### Governing equations for corrosion with stress

A continuous order parameter $\xi$ is introduced to describe the diffuse interface in the proposed phase field model. The order parameter physically corresponds to the dimensionless concentration of the metal, as $\xi =\overline{c }$. The dimensionless concentration $\overline{c }=1$ in the metal and $\overline{c }=0$ in the electrolyte solution.

In this model, we consider the order parameter’s evolution is driven by electrochemical reaction rate $r$. Thus, the driving force can be clearly divided into two parts: the interface energy and the electrode reaction. To describe the electrochemical reaction kinetics at the diffuse interface, an interpolating function ${h}^{\mathrm{^{\prime}}}\left(\xi \right)=30{\xi }^{2}{\left(1-\xi \right)}^{2}$ is introduced. Notice that the order parameter changes from one to zero for phase evolution in corrosion process. The phase evolution has a negative relationship with reaction rate $r$. Therefore, the governing equation for the phase evolution is

15 $$\frac{\partial \xi }{\partial t}={-L}_{\sigma }\left({g}^{^{\prime}}\left(\overline{c }\right)-\kappa {\nabla }^{2}\overline{c } \right){-L}_{\eta }{h}^{^{\prime}}\left(\xi \right)\left(\mathrm{exp}\left(\frac{\left(1-\alpha \right){\eta }_{a}}{RT}\right)-{\overline{c} }_{+}\mathrm{exp}\left(\frac{-\alpha nF{\eta }_{a}}{RT}\right)\right).$$

Equation (14) indicates that the mechanical deformation changes the total chemical potential with the mechanical potential ${\mu }_{me}$ (in term ${\eta }_{a}$). If ${\mu }_{me}>0$, the mechanical contribution has a positive influence on the corrosion process; if ${\mu }_{me}<0$, the mechanical contribution has a negative influence on the corrosion process.

The metal atom is considered as fixed except during diffusion process. The electrochemical reaction provides the source term which depends on the corrosion (metal consumption) process. The diffusion can be described with the Nernst-Plank equation as

16 $$\frac{\partial {\overline{c} }_{+}}{\partial t}=\nabla \cdot \left({D}^{eff}\nabla {\overline{c} }_{+}+\frac{{D}^{eff}{\overline{c} }_{+}}{RT}nF\nabla \phi \right)-\frac{{c}_{s}}{{c}_{0}}\frac{\partial \xi }{\partial t},$$

where ${D}^{eff}$ represents the effective diffusion coefficient as ${D}^{eff}={D}^{e}h\left(\xi \right)+{D}^{s}(1-h(\xi ))$, where ${D}^{e}$ and ${D}^{s}$ are the diffusion coefficients for metal cation in the electrode and electrolyte, respectively.

The mechanical equilibrium equation is expressed with stress tensor $\sigma ={A}^{e}{\varepsilon }^{eq}$ as

17 $$\mathrm{div}\left(\sigma \right)=0,$$

where the body force is neglected.

It is obvious that fully corroded metal cannot support stress or strain. The equivalent elastic strain tensor considering solid–liquid interface is modified as follows:

18 $${\varepsilon }^{eq}=p\left(\overline{c }\right)\left\{{\varepsilon }_{ij}^{e}\right\}=p\left(\overline{c }\right)\left\{\frac{1}{2}\left(\frac{\partial {u}_{i}}{\partial {x}_{j}}+\frac{\partial {u}_{j}}{\partial {x}_{i}}\right)\right\}\left(i,j=\mathrm{1,2},3\right),$$

where ${u}_{i}$ and ${u}_{j}$ are displacement components, $p\left(\overline{c }\right)$ is an interpolation function to smooth the discontinuity in the interface and it also satisfies $p\left(0\right)=0$ and $p\left(1\right)=1$. Combining Eqs. (17) and (18), we can get the governing equation for mechanical equilibrium as

19 $$\mathrm{div}\left({A}^{e}p\left(\xi \right)\left\{\frac{1}{2}\left(\frac{\partial {u}_{i}}{\partial {x}_{j}}+\frac{\partial {u}_{j}}{\partial {x}_{i}}\right)\right\}\right)=0.$$

### Pit-to-crack transition

Two different SCC initiation criteria are implemented based on the corrosion morphology and the applied mechanical load.

One criterion is based on the Von Mises yield criterion or Tresca yield criterion (which give the same results under a plane strain assumption). The crack is assumed to initiate when the stress reaches the Von Mises yield criterion. Note that this is an approximate conservative criterion for crack initiation

20 $${\sigma }_{1}-{\sigma }_{2}=2\sqrt{{\left(\frac{{\sigma }_{x}-{\sigma }_{y}}{2}\right)}^{2}+{{\tau }_{xy}}^{2}}<2Y/\sqrt{3},$$

where $Y$ is uniaxial yield stress, ${\sigma }_{y}$ is zero under a uniaxial stress state, ${\sigma }_{x}$ is the normal stress at the tip, and ${\tau }_{xy}$ is the shear stress at the tip. Both ${\sigma }_{x}$ and ${\tau }_{xy}$ at the tip position can be approximated with the stress concentration equation^35^

21 $${\sigma }_{x}={\sigma }_{norm}\left(1+2\sqrt{\frac{a}{\rho }}\right), {\tau }_{xy}={\tau }_{norm}\left(1+2\sqrt{\frac{a}{\rho }}\right) ,$$

where $a$ represents corrosion depth and $\rho$ represents the curvature at the bottom of corrosion pits or potential crack tip position. Both the curvature radius $\rho$ and corrosion depth $a$ are calculated from the corrosion morphology. From the Eqs. (20), (21), we can notice that the larger corrosion depth and sharper interfaces are more likely to initiate cracking.

**Figure 3:**
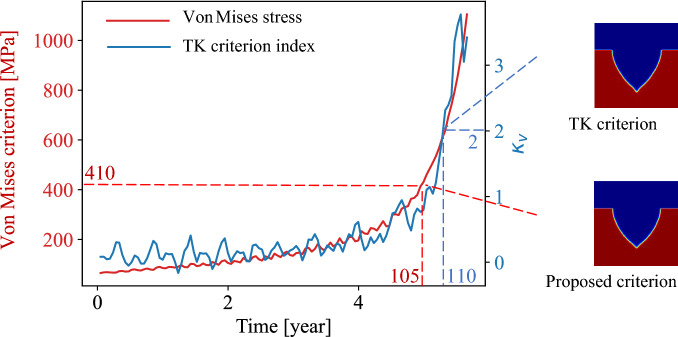
Comparation between two crack initiation criteria.

The other SCC initiation criterion is the Tsujikawa–Kondo criterion^27^, which compares the corrosion growth velocity with the crack propagation velocity. When the crack propagation speed surpasses the corrosion growth speed, the crack is assumed to initiate. One implementation is based on that the driving force from mechanical stress are separated from that from electrode dissolution^18^. The corrosion speed at pit tip and mouth area of the model are calculated as ${v}_{tip}$ and ${v}_{mouth}$ based on the displacement of the interface at pit tip location and the mouth top location, respectively. A parameter ${K}_{v}=\left({v}_{tip}-{v}_{mouth}\right)/{v}_{mouth}$ is used to evaluate the portion of driving force from mechanical stress. ${v}_{tip}$ represents the crack propagation speed and ${v}_{mouth}$ represents the corrosion speed in the crack initiation stage. When the ${v}_{tip}$ is twice as large as the ${v}_{mouth}$, implying the mechanical driving force is much larger than the corrosion without stress, the crack is assumed to initiate. Both criteria are implemented and compared as given in Fig. 3. Both the approximated Von Mises stress at the tip and parameter ${K}_{v}$ are plotted over time. The SCC initiation time is identified when the criterion is met, i.e., the Von Mises stress reaches the yield stress, or ${K}_{v}$ reaches two. The proposed SCC initiation criterion is an indicator about the time and location of a possible SCC initiation. The overall trend for these two parameters is similar while the criterion based on the approximated stress is more conservative and stable than that based on corrosion growth speed.

## Surrogate model of pitting corrosion simulation and crack initiation

The inputs for the proposed surrogate model are the corrosion morphology at a certain time step $t$, the external mechanical load, and other physical parameters including reaction constant and diffusion coefficient that would affect the growth of the corrosion pits over time. The output of the surrogate model is the corrosion morphology at future time step $t+n \left(n>0\right)$. Specifically, the corrosion morphology here are represented by the order parameter $\xi$ value in the phase-field model defined in Section “Governing equations for corrosion with stress”, ranging from 0 to 1 where 0 means liquid while 1 means metal. 80 by 40 pixels for one channel ($\xi$) are extracted from the FEM implementation results to represent the corrosion morphology in our study.

**Figure 4:**
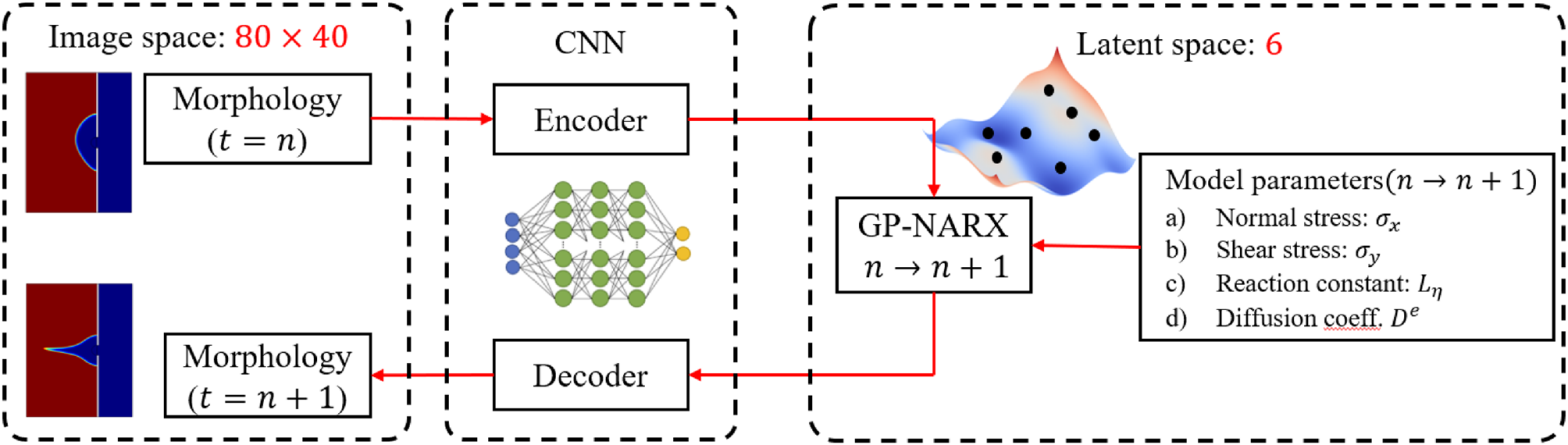
Workflow of CNN-GP-NARX Surrogate model.

To extract features, a CNN-Autoencoder and decoder is used to map the high-dimensional corrosion morphology into low-dimensional compressed features in the latent space. Low-dimensional features need to be extracted from the images to feed into the GP-NARX algorithms, as fewer features require fewer training data and less training time. The model also can make more accurate predictions with fewer features if features encode the essential information needed for prediction. Figure 4 presents the overall workflow of the CNN-GP-NARX surrogate modeling method.

### CNN-autoencoder and decoder for dimension reduction

A CNN is selected as the autoencoder in our surrogate model for feature extraction because a CNN is the most widely used architecture for image data. Several filters in the convolutional layers would extract important features when images pass through. We compared CNN with commonly used dimension-reduction techniques, such as principal component analysis (PCA). The results show that CNN has a better performance. PCA requires hundreds of principal components to obtain 98% variance for 80-by-40 pixel images, while CNN can reduce the dimension to fewer than ten with accurate reconstruction.

**Figure 5:**
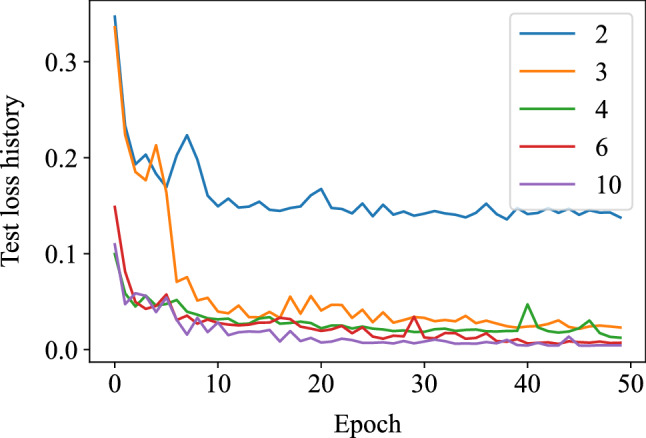
Test loss history of different numbers of features.

**Figure 6:**
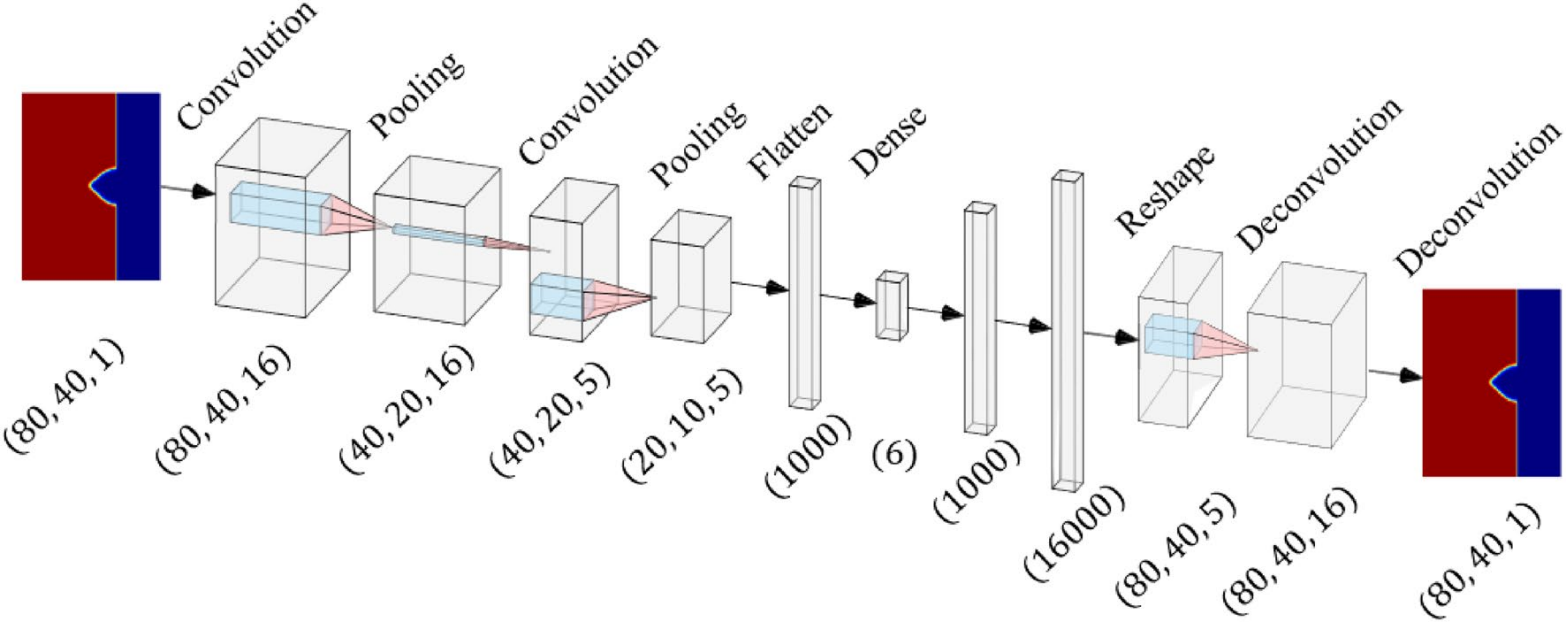
Architecture of CNN-autoencoder.

Different numbers of features have been tested for the CNN architecture and the test loss history is shown in the Fig. 5. The decrease of the test loss with the increase of the number of features stops when the feature number gets to six. This number might be sensitive to the size and architecture of the neural network. In this paper, the number of latent features is therefore chosen as six for the CNN. As shown in Fig. 6, the CNN-autoencoder contains six layers, including two combinations of one convolutional layer followed by one max-pooling layer to reduce the dimension, a dense layer to flatten multi-dimensions output from previous convolutional layers to one-dimensional vector, and a fully connected layer to reduce the length of the one-dimensional vector from the dense layer to the ideal number of features. In this network, six features are kept after compression with encoder to balance the error and dimension reduction performance according to the test results shown in Fig. 4.

The decoder consists of two fully connected layers followed by two deconvolutional layers to reconstruct the image from the compressed features. There are no un-pooling layers because that requires extra information, namely the indices of maximum values from the max-pooling layer. However, the decoder should work independently without any extra information from the encoder after training. In other words, the information needed in the images is not properly compressed into features since there are still some information that lies in the indices of maximum values.

The encoder and decoder are trained together. The purpose of encoder-decoder structure is dimensionality reduction. Therefore, more accurate reconstruction results are desired from low-dimensional latent space. Thus, the input and output for training is the same image. In this case, 75% of the morphology images are randomly selected for training, and the remaining 25% is used to validate the neural network.

### GP-NARX models

Gaussian process regression (GPR) is a probabilistic predictive model class that has been widely used in many domains to build a data-driven model for prediction^36^. It has an advantage over other machine learning models in that it provides not only mean predictions (a “point estimate”, like a neural network) but also confidences in a closed form. While GPRs are widely employed for surrogate modeling of static and quasi-static problems, they may be extended for dynamic systems by adopting a nonlinear autogressive exogenous model (NARX) framework. NARX model describes the relationship between the responses ${y}_{i}$ at ${t}_{i}$ as a nonlinear function of exogenous inputs as follows

22 $${y}_{i}=G\left({y}_{i-1}, \cdots , {y}_{i-p}, {u}_{i}, \cdots , {u}_{i-q} \right)+{\varepsilon }_{i},$$

where $G(\cdot )$ is a nonlinear function, ${u}_{i}, \cdots , {u}_{i-q}$ are the exogenous inputs, *p* and *q* are respectively the number of lags for the inputs and the response variable, and ${\varepsilon }_{i}$ is the error term.

In GP-NARX, a GPR model is constructed to learn the nonlinear function $G(\cdot )$. Let the latent responses of corrosion morphology after the mapping using CNN-autoencoder be ${{\varvec{\upalpha}}}_{1}, \cdots , {{\varvec{\upalpha}}}_{6}$ , where ${{\varvec{\upalpha}}}_{j}=\left[{\alpha }_{j1}, {\alpha }_{j2}, \cdots ,{\alpha }_{j{N}_{t}}\right],\forall j=1,\cdots , 6$ is a vector of the *j*-th latent response, ${\alpha }_{jk}$ is the *j*-th latent response at time step ${t}_{k}$, and ${N}_{t}$ is the total number of time steps in the pitting corrosion simulation, six GP-NARX models are constructed in the latent space as follows

23 $${\alpha }_{jk}={\widehat{G}}_{j}\left({\widetilde{{\varvec{\upalpha}}}}_{1}, \cdots , {\widetilde{{\varvec{\upalpha}}}}_{6}, {\varvec{\uptheta}}\right)+{\varepsilon }_{k},\forall j=1,\cdots , 6,$$

in which ${\widetilde{{\varvec{\upalpha}}}}_{j}=\left[{\alpha }_{j1},\cdots ,{\alpha }_{jp}\right], \forall j=1,\cdots , 6$ are the previous *p* time steps of the *j*-th latent response, ${\alpha }_{jk}\sim N({\mu }_{jk}, {\sigma }_{jk}^{2})$ is a probabilistic prediction which is represented as a Gaussian distribution with mean ${\mu }_{jk}$ and standard deviation ${\sigma }_{jk}$ from the GP-NARX models, and ${\varvec{\uptheta}}$ is a vector of other input parameters that affect corrosion growth at previous time steps. Note that parameters ${\varvec{\uptheta}}$ change slowly in the corrosion problem and thus are assumed to be constant in the simulated time period.

Once the GP-NARX models are trained, there are two kinds of prediction that may be performed. The basic one is one-step ahead prediction. One-step ahead prediction is made with the observations from previous certain steps, which is using morphology features of a few continuous time steps to predict latent features of the following step, i.e.,

24 $${\alpha }_{jk}^{*}={\widehat{G}}_{j}\left({\widetilde{{\varvec{\upalpha}}}}_{1}, \cdots , {\widetilde{{\varvec{\upalpha}}}}_{6}, {\varvec{\uptheta}}\right)+{\varepsilon }_{k},\forall j=1,\cdots , 6;\mathrm{where}\, {\alpha }_{jk}^{*}\sim N\left({\mu }_{jk}^{*}, {\sigma }_{jk}^{*2}\right).$$

The other kind of prediction is called multi-step ahead prediction. In multi-step ahead prediction, the prediction at the current time step will be used as inputs of the future time steps and is performed recursively as follows

25 $${\alpha }_{jk}^{*}={\widehat{G}}_{j}\left({\widetilde{{\varvec{\upalpha}}}}_{1}^{*}, \cdots , {\widetilde{{\varvec{\upalpha}}}}_{6}^{*}, {\varvec{\uptheta}}\right)+{\varepsilon }_{k},\forall j=1,\cdots , 6,$$

where ${\widetilde{{\varvec{\upalpha}}}}_{j}^{*}=\left[{\alpha }_{j1}^{*},\cdots ,{\alpha }_{jp}^{*}\right]$ is a vector of uncertain predictions from previous time steps. Usually, multi-step ahead prediction is more demanding for the model, as the errors from every previous prediction can accumulate.

Since a large volume of data may be generated from each simulation in the pitting corrosion simulation process, each simulation data step is down-sampled by every third time step, as the original time interval is too small to show obvious corrosion evolution. After the data down-sampling, *p* = 5 is chosen in this paper, which is a number determined through cross-validation that can give the best prediction. It means that the compressed morphology features of previous 5-time steps (15-time steps from the simulation) are used in the input of NARX structure. The considered model parameters ${\varvec{\uptheta}}$ as mentioned above include external normal load, external shear load, reaction constant, and the diffusion coefficient.

### Uncertainty propagation

The above presented CNN-GP-NARX based surrogate modeling method is hundreds of times faster than the traditional high-fidelity simulation of pitting-to-cracking process. In order to compare prediction performance to the original high-fidelity simulation, it is necessary to quantify the uncertainty in the surrogate model prediction. In this paper, the uncertainty quantification of surrogate model prediction is computed directly using Monte Carlo simulation (MCS) since the constructed surrogate model can run very efficiently. In MCS, a large number of MCS samples are first generated for the initial steps of the GP-NARX models. The initial samples are then propagated to the latent responses at later time steps, by following the NARX framework discussed above. The generated latent-space samples at each time step are then transformed back to corrosion morphology images using CNN autoencoder. For each corrosion morphology image, the crack initialization behaviors are analyzed using the method presented in Section "Governing equations for corrosion with stress". Based on that, we obtain statistical information about whether, when, and where the corrosion damage triggers the crack initiation.

## Results and discussions

### Calibration of the phase-field model

**Figure 7:**
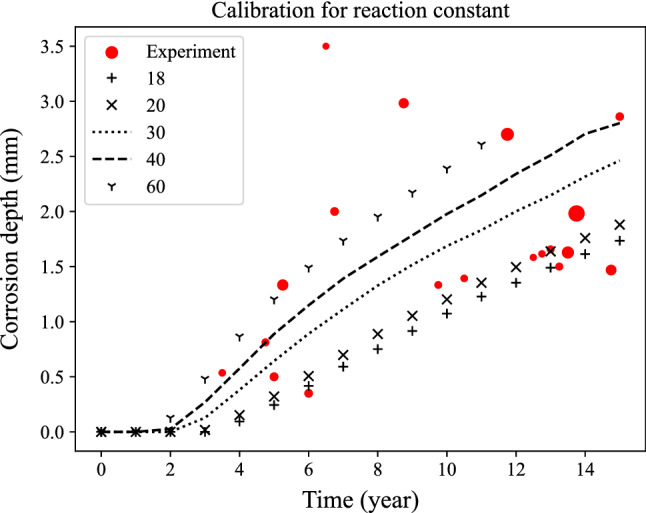
Calibration of reaction constant with measurement data^37^.

The electro-chemo-mechanical phase-field model is calibrated through the measurement data in the literature^33,37,38^. In the phase field model for a new system, the reaction constant ${L}_{\eta }$ is usually unknown or unavailable. Here, ${L}_{\eta }$ is calibrated through the measurement of the inner bottom plates of sea-going bulk carriers as shown in Fig. 7. The measured data is not stable because there were some repair and replacement actions going on during the measurement^37^. The different size of the spots represents the number of pits with a certain depth at a certain time. We can notice that changes in the reaction constant greatly affect the corrosion growth. The size of the measurement data point represents the number of pits at certain time with certain depth. The value 40 after normalization, corresponding to $1.3\times {10}^{-7}/s$ is selected for ${L}_{\eta }$ in this work. Details about the normalization of all parameters can be found in the appendix. Other parameters such as gradient energy coefficient and diffusion coefficient are chosen from the literature^33,38^.

### Stress effect on corrosion

**Figure 8:**
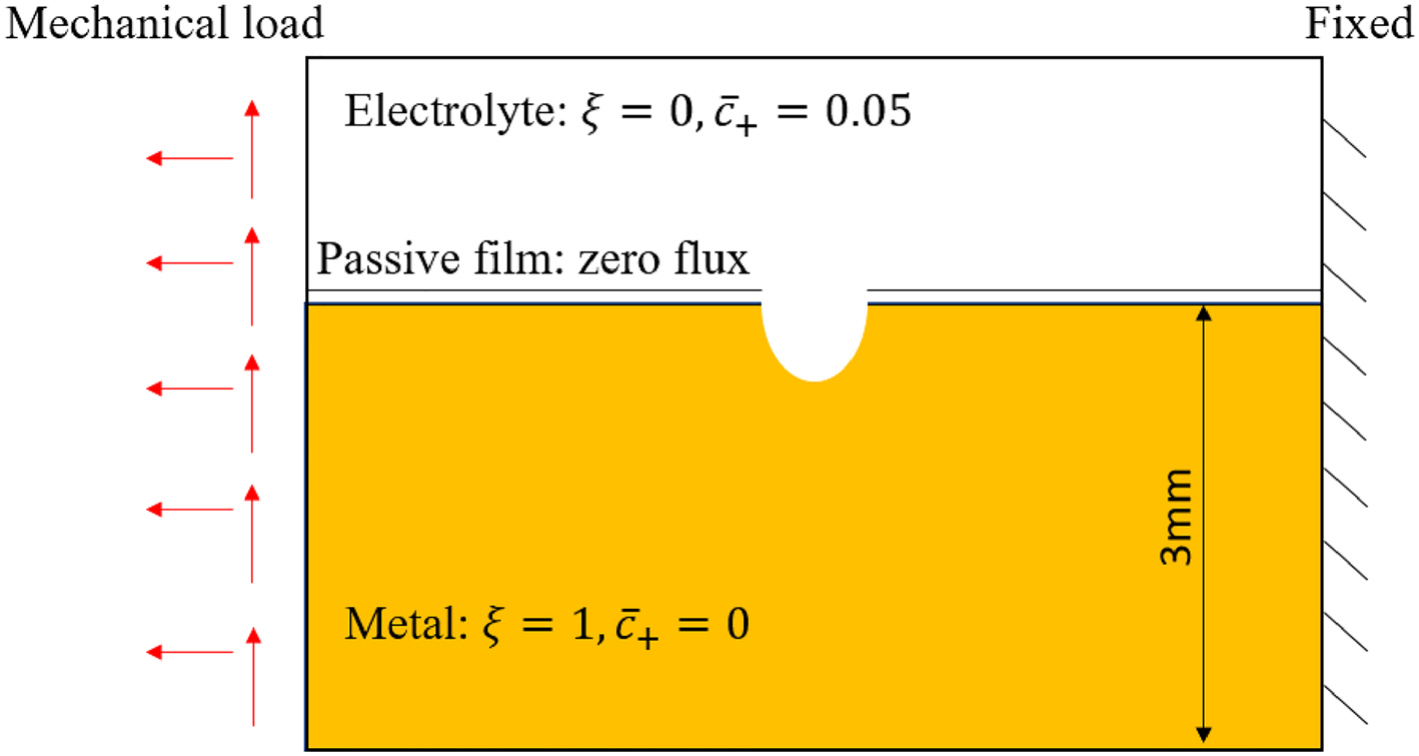
Geometry and boundary conditions of the model for stress effect study.

**Figure 9:**
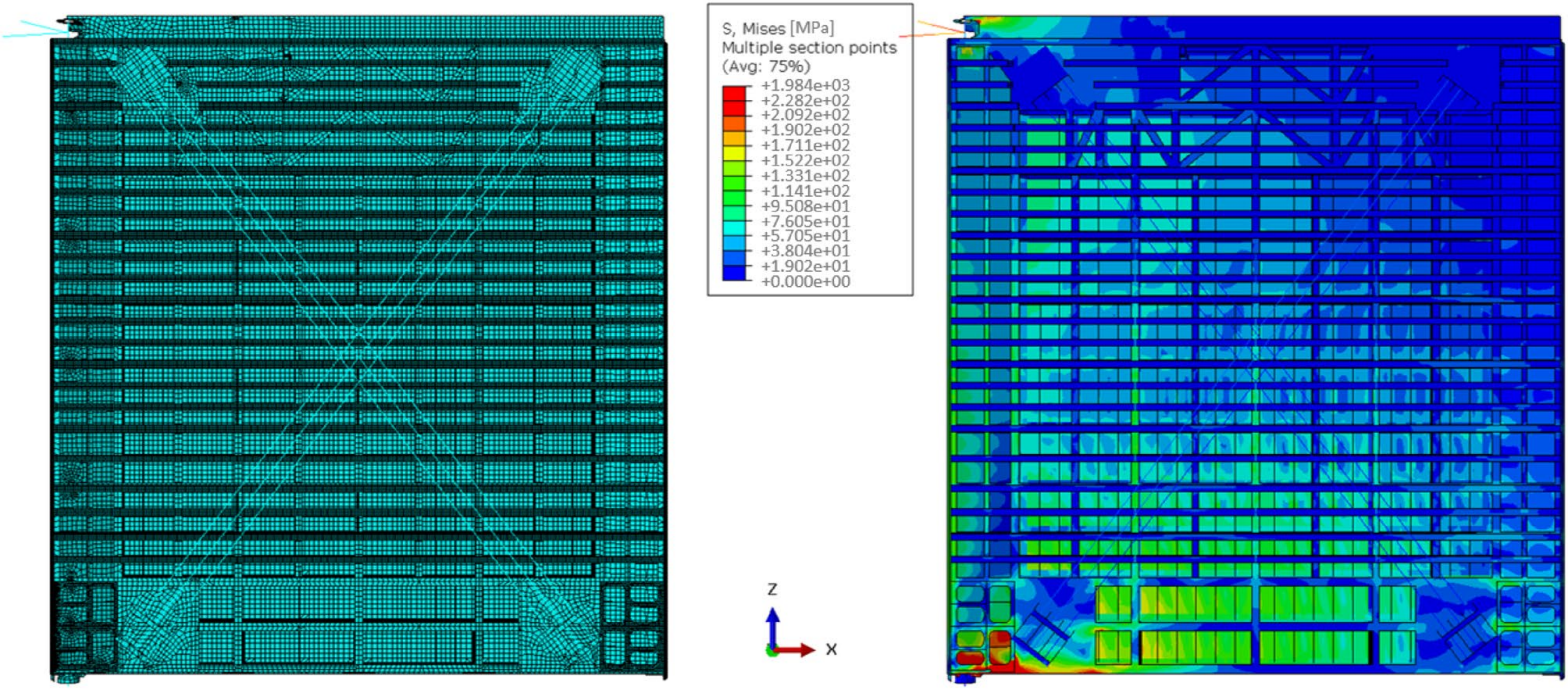
Stress magnitude of miter gate simulation.

The influence of different kinds of mechanical loading on corrosion evolution is considered in this section. Figure 8 shows the geometry and boundary conditions of the model. The interface between metal and electrolyte is separated with a layer of non-penetrable passive film except the center semicircle area, which is the initial pit assumed. Normal stress, shear stress with an average magnitude of 50 MPa, and combinations therein are applied to the model because these stress status are common in a miter gate (a type of large civil infrastructure that serves as the underlying structure of interest in this work) in service according to a validated simulation^39^ as Fig. 9 shows. The right surface is fixed. Plane strain is assumed for the model.

**Figure 10:**
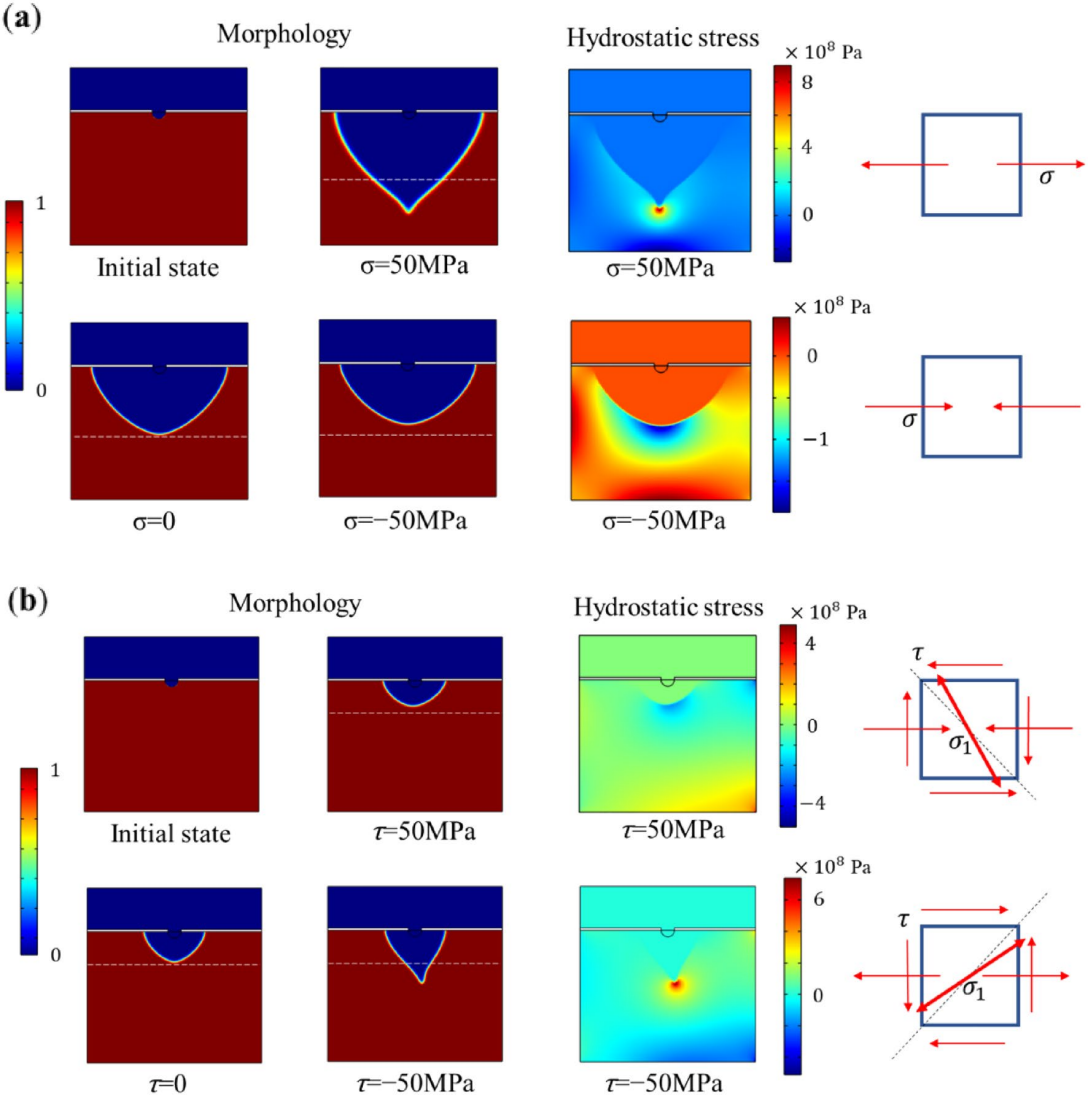
Results of pitting corrosion evolution with the influence of (**a**) normal stress, and (**b**) shear stress.

Figure 10a compares the influence of constant normal tensile stress and compressive stress on the corrosion process. Given applied stress, tensile stress leads to greater corrosion depth while compressive stress results in less corrosion depth. The morphology shows that the tensile stress results in a sharper interface while compressive stress results in a flatter interface. Both cases with tensile stress and compressive stress have stress concentrations because of material loss in the pit evolution. Note that the stress condition affects the corrosion evolution with the hydrostatic stress term in the mechanical elastic energy density as indicated in Eq. (2). As the corrosion evolves, the absolute value of hydrostatic stress at corrosion tip increases greatly since stress concentration is severe. Therefore, the contribution of mechanical stress is becoming more and more dominant as the stress concentration increases. However, the tensile stress leads to positive hydrostatic stress while compressive stress leads to negative hydrostatic stress. Large positive hydrostatic stress in the tensile case accelerates the corrosion growth. Because stress concentrates at the pit tip, the tip area has larger positive hydrostatic stress. Thus, corrosion in this area grows faster than in neighboring areas such that the interface becomes sharper. A sharper interface leads to more serious stress concentration. This cycle accelerates the corrosion evolution and fosters potential crack initiation. Conversely, in the compressive case, the negative hydrostatic stress decreases the corrosion growth. As the absolute value of the negative hydrostatic stress increases at the tip area due to stress concentration, the corrosion evolves slower than in other areas. This leads to a flatter interface as the morphology figure shows.

Figure 10b shows the influence of constant shear stress on the corrosion evolution. Positive and negative shear stress are applied to the model. Negative shear stress leads to larger corrosion depth while positive shear stress leads to smaller corrosion depth than the zero-stress case. In addition, negative shear stress also changes the corrosion (or SCC) evolution direction. Negative shear stress applied on the top surface brings tension stress to the right side of the model. The tension stress leads to positive hydrostatic stress, contributing to faster corrosion growth. The sharp interface is formed with similar reasoning as with tensile stress case discussed above. The change of corrosion evolution direction is interesting. The shear stress acting on a stress element can result in a “shear diagonal”, which is the principal tension in the diagonal direction as shown in Fig. 10b. This principal tension creates higher hydrostatic stress in the perpendicular direction. With tensile stress on vertical direction, the principal stress direction is smaller than 45 degrees. Therefore, the corrosion evolves with the perpendicular direction to the principal stress direction.

However, the positive shear stress case shows completely different behavior. This is because positive shear stress at the top surface leads to compressive stress, and thus negative hydrostatic stress to the right side of the model. This results in a slower corrosion depth growth. Similar to the negative shear stress case, positive shear stress also results in shear diagonal at the other diagonal direction. There is slightly more corrosion (upper left side) on the perpendicular direction to the shear diagonal. The difference is not as obvious as the negative shear stress case, as the compressive stress decreases the overall corrosion growth, and thus there is less stress concentration reducing the driving force from mechanical stress.

**Figure 11:**
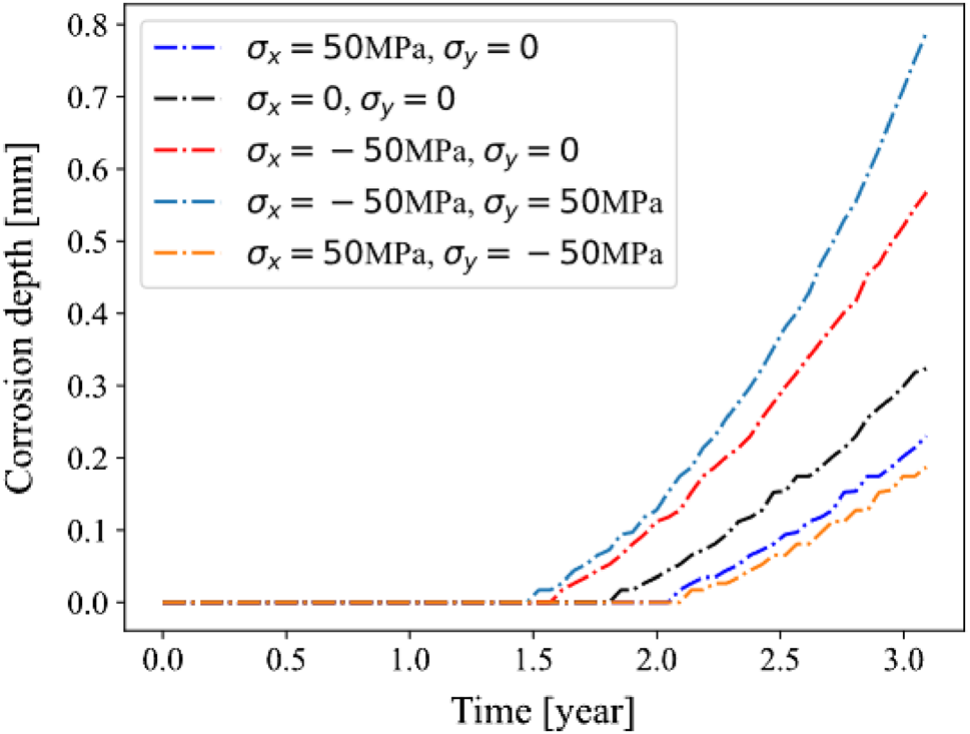
Corrosion depth under different stress conditions.

Figure 11 shows the effect of combined mechanical load of normal and shear stress. We can find that a tensile stress with a negative shear stress leads to the fastest corrosion depth growth, while a compression stress with a positive shear stress leads to the slowest corrosion depth growth. This is because the complex stress can result in higher or lower hydrostatic stress in the model.

**Figure 12:**
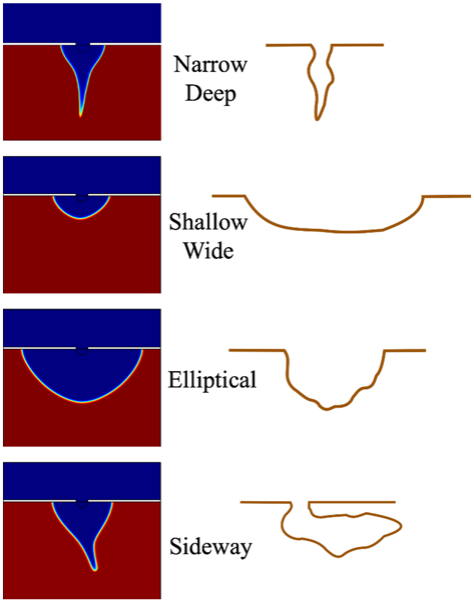
Different pitting shapes from simulations (left) and observations (right).

It should be noted that pitting corrosion/propagation is a complex process that involves multiple chemical reactions, environmental impacts, and different kinds of nucleation mechanisms. This part mainly focuses on the influence from mechanical stress on the pitting corrosion. Therefore, the pitting shapes from this model cannot cover all the shapes reported from experimental observation^40^. However, some shapes are still foreseen from shapes in this model. As the Fig. 12 shows, when the metal is under tensile loading, the pitting shape tends to be sharp and narrow; when under compression, the shape becomes wide and shallow; when without mechanical actions, the shape becomes semi-circle like, indicating isotropic corrosion; when under certain direction shear stress, sideway pit is more likely to be formed. In the future, the model can be extended with the environmental conditions associated with nucleation mechanisms as well as material microstructures.

### Stress effect on the SCC initiation

**Figure 13:**
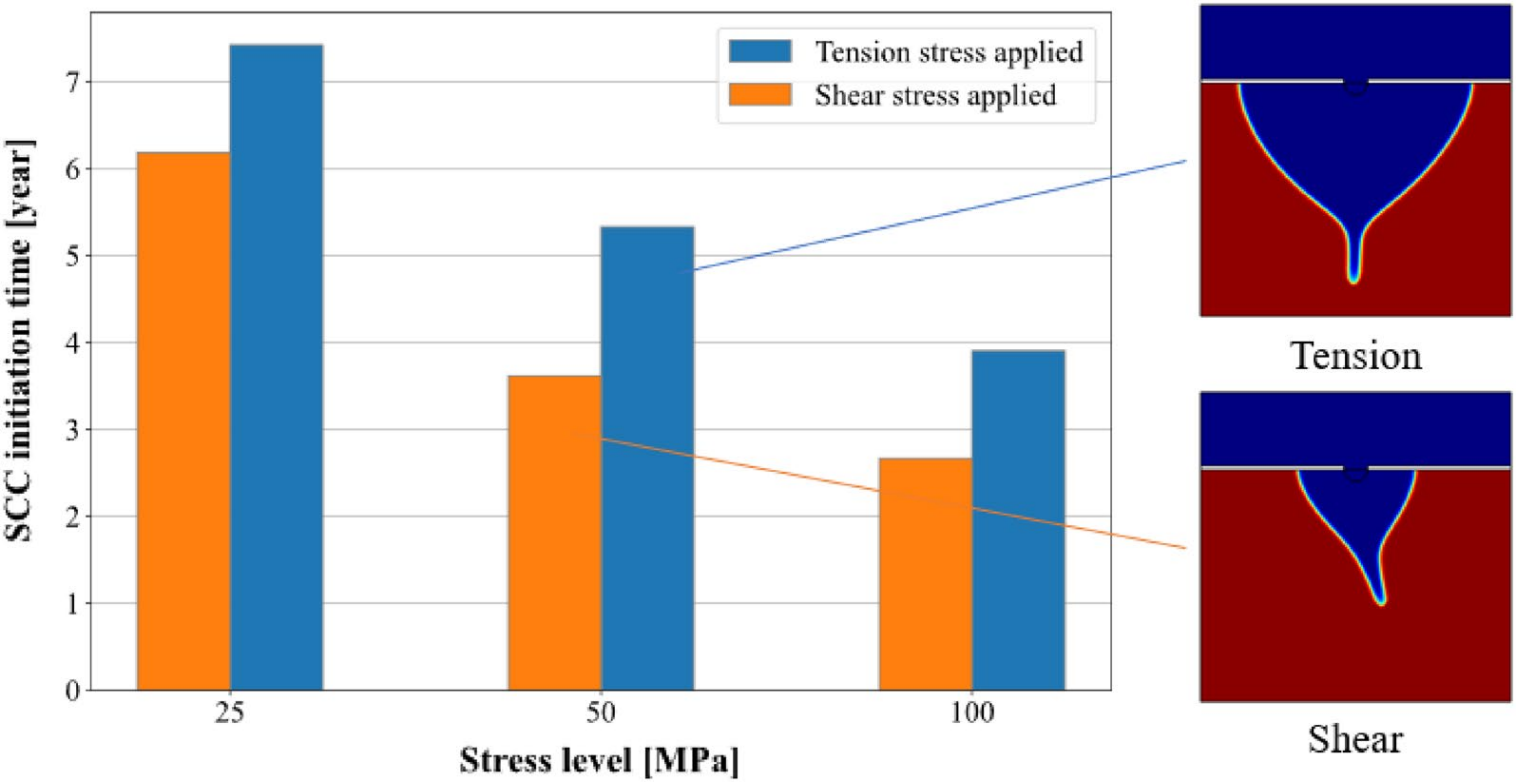
Stress influence on SCC initiation.

Different normal stress and shear stress conditions are applied to the model to investigate the influence of stress level on the SCC initiation time. There is no SCC initiation when the compressive stress and positive shear stress are applied because they decrease the corrosion growth speed at the tip location where the stress concentration is critical; however, tensile stress and negative shear stress can lead to SCC initiation as Fig. 10 shows. For both tensile stress and shear stress, the SCC initiation time decreases with the increase of stress level, which means higher stress level leads to an earlier SCC initiation. For the same magnitude of stress, shear stress results in earlier SCC initiation than tensile stress. The morphology on the right of Fig. 13 also shows the SCC propagation direction of both the tension case and the shear case corresponding to the crack direction in the tension and shear failure modes.

### Surrogate model results

#### Data preparation

Due to the lack of availability of measurement data and the challenges associated with creating long-term corrosion experiments (which is beyond the scope of this initial study), all data used for training and validating the framework is generated by the proposed calibrated high-fidelity phase-field model. The dataset used to train a surrogate model needs to seek as much information as possible with a moderate computational cost.

Four parameters—external normal stress, external shear stress, reaction constant, and diffusion coefficient—are chosen as the input parameters to the surrogate model. External load is the mechanical load applied to the top and bottom surface of the plate structure, which is tensile stress or compressive stress in the model. The reaction constant describes the rate of electrochemical reaction in the system. The diffusion coefficient represents the ratio of flux density to the negative of the concentration gradient in direction of diffusion. These four parameters are considered as major driving factors for the corrosion process. The ranges of chosen parameters are determined based on the calibration results given in Section "Calibration of the phase-field model". Then, a Sobol sampling technique is used to obtain a low discrepancy quasi-random sequence of the model parameters, which are inputs of the training data. For each training data set of inputs (external load, reaction constant, and diffusion coefficient), 200-time steps of the high-fidelity phase-field simulation are executed, and 200 corrosion morphology images are obtained. In total, 51,000 corrosion morphology images are generated from the 255 simulations.

The whole model is overall complex and deep learned, such that more simulations for the train and validation is desirable. Given that each simulation costs more than 40 min on our workstation, 255 simulations are completed in total. 10 random selected simulations are used as the final test set for the whole surrogate model, combining CNN-autoencoder and GP-NARX to check the multi-step prediction ability in the image space. This set has never been presented to the neural network before testing. They are in neither the encoder-decoder training process, nor the GP-NARX training process.

The CNN-autoencoder part is trained with randomly selected 90% of total 245 $\times$ 200 images and validated with the rest 10%. The data dimension after compression is $6\times 245\times 200$. As explained in Section “GP-NARX models”, the features are down-sampled by every third time step, leading to a $6\times 245\times 66$ dimension. The GP-NARX model predicts the features of the next time step based on the 5 previous time steps. Therefore, the data are sliced into multiple 6-time-steps pieces where the first five serve as a part of the input of the GP-NARX model and the last serves as the output. 90% of slices are randomly selected as the training set and the rest are for the validation. There is still little overlap for the training and validation sets in the encoder-decoder part and GP-NARX part because the GP-NARX models are dynamic models while the CNN-based encoder-decoder model is static.

#### Low dimension features representation of phase-field results

**Figure 14:**
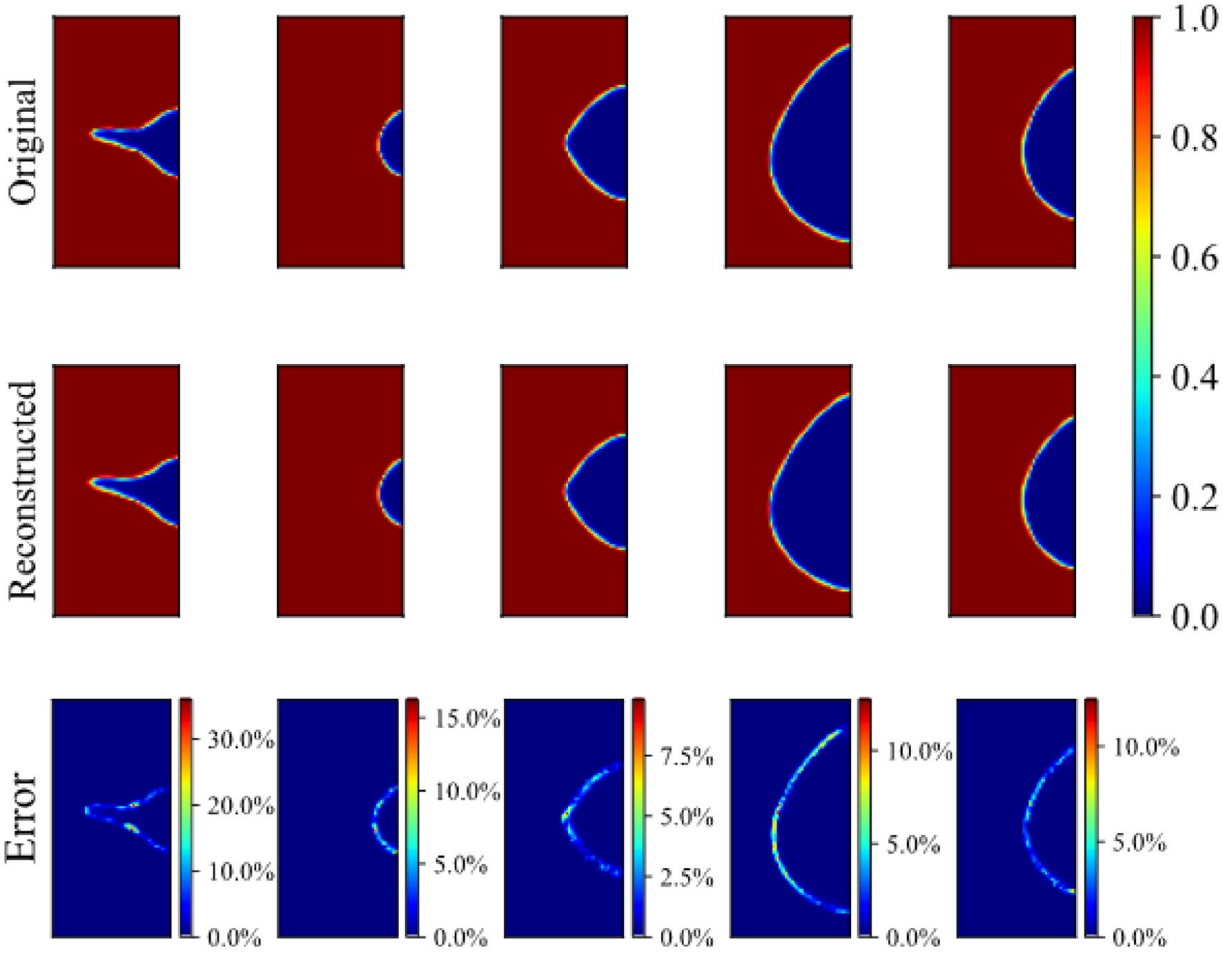
Comparison and error of reconstructed figure from latent space.

A CNN-autoencoder is trained together with the decoder. Images with 80 by 40 pixels are compressed with the autoencoder to only six features in the latent space. Then the six features are used to reconstruct the original images. The dimensionality reduction rate which is calculated as the dimension after compression divided by the dimension before compression, is about 0.19%. The encoder decoder model is later tested on a validation set which is not seen by the model. Figure 14 shows the results comparison between the original images and the reconstructed images.

Although the dimension is reduced significantly with a dimensionality reduction rate less than 1%, the overall error due to dimension reduction is negligible. The first row shows the original corrosion morphology from different time step results of dynamic phase-field model. The second row show the images reconstructed with the six features after encoder. The third row shows the error between the original images and the reconstructed images as well as the error percentages. We can notice that the overall error value is close to zero in the area. The most error concentrates on the interface area, as regions away from the interface are constant (either zero or one) while the order parameter changes between zero and one in the interface. The encoder-decoder model can accurately capture the constant regions and has good ability to compress the changing regions. Notice that the highest-error region is only a very small fraction of the whole surface.

A sharper interface would lead to larger error in the interface. For a flat morphology, the maximum error at the interface is within ten percent. For sharper interface, the maximum error at the interface is about 30 percent. One possible reason for this is that there are more flat morphology images than sharp morphology images. The dynamic corrosion results are extracted from the phase-field simulation at each time step and each time step has the same length. During the corrosion evolution process, the sharp interface lasts for very short time while flat interface lasts for a long time. This is also related to the crack initiation that will be discussed later. Therefore, there would be far more images with a flat interface than that with sharp interface. Given more training data for flat interfaces, less error for flat interface can be explained. The other reason is that a sharp morphology is more complex than that of flat interface. As a result, there is more error for more complex image, if both are compressed to the same number of features.

#### GP-NARX prediction results

Like CNN-autoencoder, ten percent and ninety percent data from the 245 simulations are served as training and validation datasets. The GP is trained with down-sampled data. Each input contains features from the previous five time steps with six dimensions in the latent space for each time step and the four parameters. Therefore, the dimension of the inputs for each GP-NARX model is 34 in total. Each output contains the features for the next step, which is six dimensions. Additional ten simulations data is used as the testing dataset.

**Figure 15:**
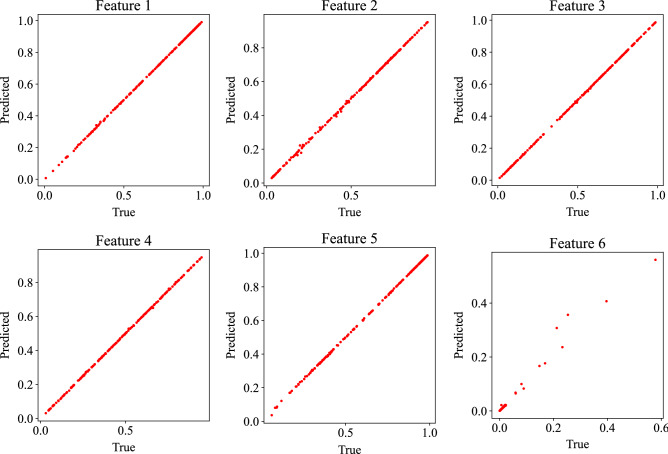
One step ahead prediction results in the latent space.

Figure 15 shows the result of one-step ahead prediction of the trained GP-NARX models. Random samples from validation set are predicted and the prediction results are plotted with true values. We can see that the prediction is very accurate for features 1 to 5. The predicted value and the true value are very close, and the points are distributed more uniformly. Most samples of feature 6 concentrate on small values near zero. Most values are within 0 and 0.03. Such small change might indicate that the feature 6 is not making a significant difference most of the time but there are some large values exists as well. Some predictions are slightly off the diagonal line, but overall, the prediction is still accurate. To further evaluate the performance of the model, the more challenging multi-step ahead prediction is also implemented. True feature values of the first five steps in the down sampled data and four parameters are used as the initial input and then the predicted data is used as the part of input to predict the features of the following time step. This equates to predicting sixty steps with the first five steps in the down-sampled data. To account for the uncertainties in the GP model, the predicted mean (i.e., ${\mu }_{jk}$ in Eq. (24)) is not directly used as part of the input for the next step. Instead, samples of a Gaussian distribution with predicted mean and variance are used as the part of the input for the next step. 1000 realizations are predicted with the same initial input for each simulation in the testing dataset.

**Figure 16:**
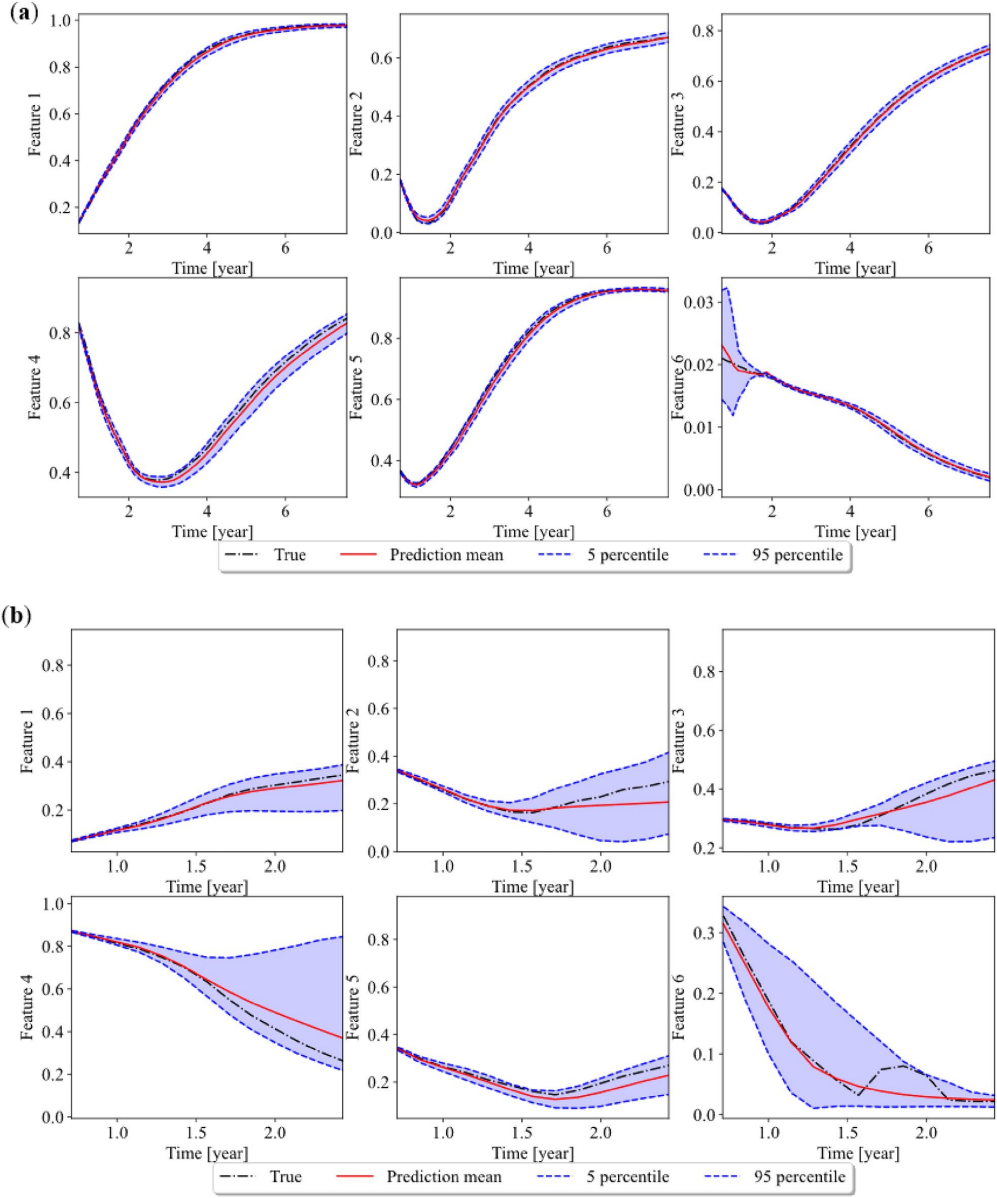
Multi-step ahead prediction result of case (**a**) and (**b**) in the latent space.

Two different cases are chosen to plot as the representative of the testing dataset. The input parameters for case (a) and case (b) of the phase-field model are 19.14 and -30.86 MPa for the shear stress, 9.18 and 34.18 MPa for normal stress, 7.13 and 2.23 for reaction constant, and 2.06 and 4.46 for diffusion coefficient, respectively. Figure 16a,b show the results of multi-step prediction of six features after compression in case (a) and case (b). We can see that the prediction is accurate for all six features for case (a). The narrow confidence interval shows that the model is confident about the prediction. Although the mean value of prediction for feature 4 slightly deviates from the true value, the true value is still within the 90 percent confidence interval. One interesting observation is that certain level of scatter is found before 2 years and getting smaller after 2 years for feature 6. This is probably because there is larger prediction uncertainty in the region before 2 years than its counterpart after 2 years in the trained GP-NARX model. At the beginning of the prediction, the model might not be so confident given such small values of inputs in that region. As the values continue to feed in, the prediction enters a region where the model is more accurate and with less uncertainty.

Figure 16b shows the prediction results for case (b). The mean prediction is not so accurate after 1.5 years. The confidence interval is wider than the prediction of case (a), which indicates that there is larger uncertainty in the prediction. During the multiple-step prediction, the error of prediction at single step can propagate to the next step and accumulates to larger error. Given there are more uncertainties in the prediction, the uncertainties accumulate resulting in large confidence interval. Although the mean of prediction for case (b) is not as precise as case (a), the true value is still within 90 percent confidence interval, which indicates that the GP-NARX model can effectively capture the uncertainty in the prediction. There is also some non-linearity after 1.5 years for feature 6 and our model does not capture it properly. This might be related to the distribution of feature 6 as most numbers concentrates on small values less than 0.03 as described in Section “GP-NARX prediction results”. Therefore, there are few samples in the range of 0.03–0.1 in the training set, or even the whole data set, leading to a poor prediction for the non-linear pattern in this range.

The difference between prediction results of case (a) and case (b) also shows the importance of quantifying the uncertainty of the surrogate model prediction, since it is difficult for a machine learning model to accurately predict the response for all regions. Based on the uncertainty quantification of the machine learning model prediction, we can then adaptively refine the surrogate model to ensure it is accurate in important regions.

#### Mapping GP-NARX predictions to corrosion morphology

**Figure 17:**
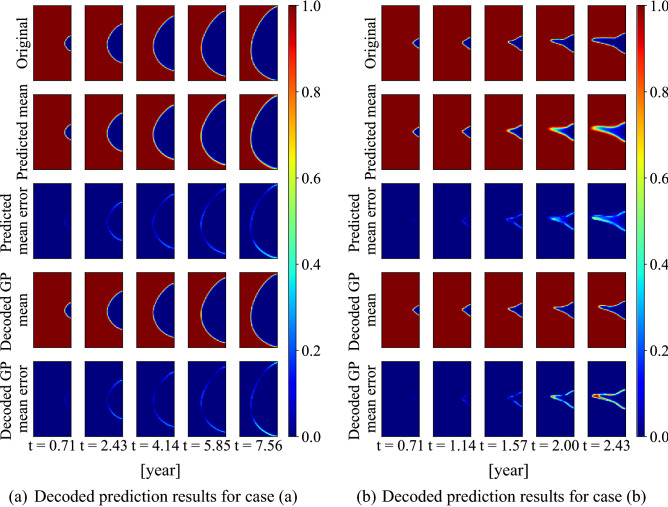
Multiple-step prediction result of the corrosion morphology.

The predicted results by the GP-NARX models in the latent space then are transformed back to corrosion morphology images using the CNN decoder. Two types of mean predictions are considered. For the first type, the surrogate model prediction uncertainty is considered in the prediction. 1000 realizations of corrosion morphology images for case (a) and case (b) are obtained from decoder at each time step. The mean of each pixel is then computed based on the uncertainty propagation and is plotted. It is referred to as the predicted mean. For the second type, the uncertainty in the surrogate model prediction is not considered. The mean (${\mu }_{jk}$) of GP-NARX model prediction is directly decoded and plotted. This is referred to as the decoded GP mean. Since the decoder is a nonlinear transformation, there is a difference between the two results. Figure 17 shows the accuracy comparison with the underlying true values for the two studied cases.

The results show that prediction results of the proposed CNN-GP-NARX surrogate model are accurate. Like the GP-NARX prediction results in the latent space discussed above, the overall prediction for case (a) after decoder remains very accurate. The errors for both predicted mean and decoded GP mean are small. For case (b), the overall prediction is still accurate. The general morphology and corrosion area can be captured. But both the predicted mean and decoded GP mean slightly underestimate the evolution of the corrosion when the morphology gets sharp, and the corrosion depth grows rapidly. Furthermore, we can notice that the prediction mean is more accurate than the decoded GP mean from the error plot. This indicates that the error of the GP model mean prediction can be compensated by the GP model variance prediction through uncertainty propagation and the nonlinear transformer, decoder. Overall, the surrogate model can give accurate predictions for different corrosion morphologies and corrosion area.

**Figure 18:**
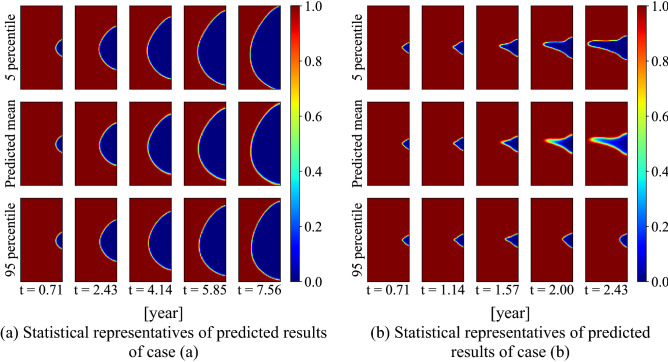
Multiple-step prediction result distribution in the image space.

Figure 18 plots the 5-th percentile and 95-th percentile images obtained from the 1000 corrosion morphology images decoded from the latent space after uncertainty propagation. For case (a), the 5-th percentile, mean and 95-th percentile are quite close to each other. This corresponds to a high confidence in the GP prediction result in the latent space. For case (b), the 5-th percentile shows the largest corrosion area as well as the corrosion depth with a similar corrosion morphology as the mean. The 95-th percentile shows the least corrosion area and the corrosion depth with a different morphology. This is also consistent with the wide confidence interval in the GP results in latent space. The comparison also shows the uncertainty (wide confidence interval) in the GP prediction results represent different corrosion evolution processes, i.e., different corrosion evolution speed with different corrosion morphologies. The uncertainty propagation process can thus reasonably capture the uncertainty in the prediction, as evidenced that the true value is contained in the prediction confidence interval.

**Figure 19:**
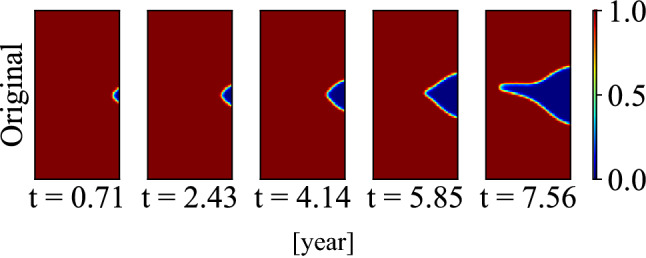
Case (c) morphology evolution.

**Figure 20:**
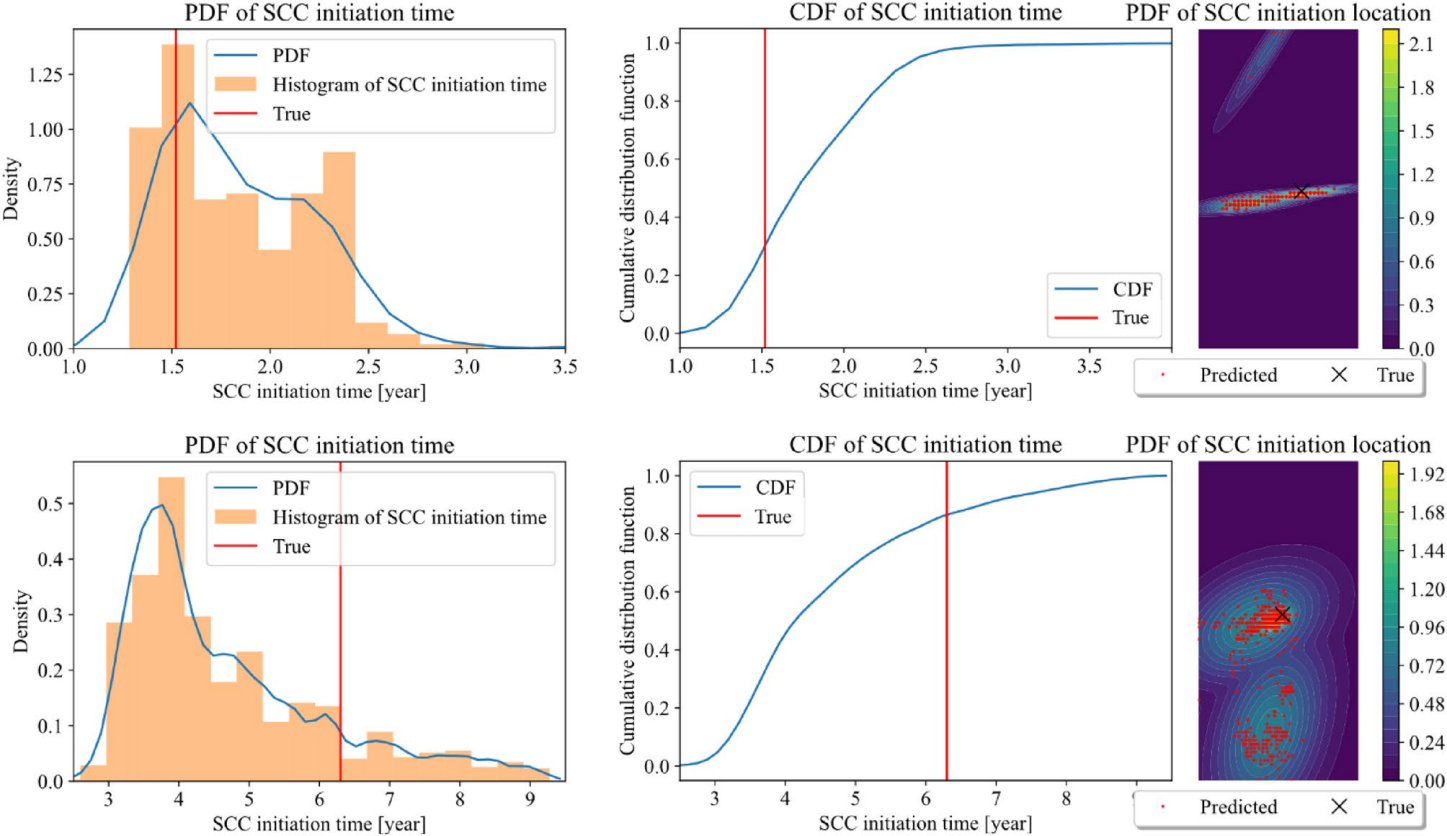
SCC initiation time and location distribution for case (b) given in Fig. 17 and case (c) given in Fig. 19.

Stress based SCC initiation criterion is applied to further propagate the uncertainty in the corrosion morphology prediction to SCC initiation time and location. To show the SCC initiation prediction for different cases, case (c) as Fig. 19 shows is added as there is no SCC initiation in case (a). The input parameters of the phase-field model for case (b) and case (c) are -30.86 and -24.61 MPa for the shear stress, 34.18 and 12.30 MPa for normal stress, 2.23 and 0.39 for reaction constant, and 4.46 and 1.76 for diffusion coefficient, respectively. Figure 20 shows the probability density function and cumulative density function of the crack initiation time and location for case (b) and case (c).

The prediction of SCC initiation time and location is accurate for case (b) as the predicted values with the largest probability densities are very close to the true value. For case (c), the predicted SCC initiation time is conservative while the predicted location is still accurate. We can find that the average SCC initiation time is earlier than the true value for case (c). Comparing the distributions of case (b) and (c), it may be seen that the distribution of SCC initiation time is more concentrated for the case (b). One reason could be that and the reaction constant and the stress acting on case (b) is larger than that of case (c), resulting a faster corrosion rate. When the corrosion speed is faster, the potential SCC initiation and propagation is also faster. Therefore, the time range of SCC initiation time is also smaller in the faster case, i.e., the SCC initiation time is more concentrated for case (b) than case (c) in MCS. The SCC initiation location distribution shows that the SCC initiation location is also more concentrated at certain area for case (b) than case (c).

## Conclusions

A computational framework for probabilistic analysis of corrosion-to-crack transitions is developed and explored. The proposed surrogate model can accurately predict the corrosion evoluation and the time as well as the location of potential SCC initiation when there is mechanical stress acting on a structure. The proposed sorrogate modeling method reduces the computational cost significantly, which enables much more rapid predictive assessments. It also accurately captures the interface, which is the material boundary in the corrosion process. The uncertainty in the surrogate model is propagated to crack initiation time and location, such that the distribution of crack initiation time and location is calculated. The proposed framework can also consider other uncertainty sources in the future.

The influence of mechanical stress on the corrosion evolution and SCC initiation is analyzed in details. Tensile stress results in faster corrosion speed than compressive stress. Complex stress case can cause faster or slower corrosion speed than any simple stress of the same magnitude. Both tensile and shear stress can lead to sharp interface and potential crack initiation. Larger stress and higher reaction coefficient induce earlier crack initiation time. There are also some limitations in the current corrosion model. The impact of the environment is not studied in this paper. The parameters related to the environment (such as reaction constant, diffusivity of metal ion) are considered as constant. The corrosion nucleation mechanism is not considered in this paper. The corrosion is assumed to start at the beginning of the simulation. In addition, pitting corrosion/propagation is a complex process that involves multiple chemical reactions and affected by different microstructures of the metal. The predicted pitting morphologies in this work cannot cover all the pitting formations reported in experimental observation ^40^. These issues will be studied in our future work.

## Data Availability

Simulation data used to formulate this study’s results, which total more than 50 GB, may be made available upon a reasonable request to the corresponding author, or the corresponding author’s delegate.

## Appendix

### Normalization table

**Table:**

Parameter	Real value	Normalization	Normalized Value
Reaction constant ${L}_{\eta }$	$8.3\times {10}^{-6}/\mathrm{s}$	${\widetilde{L}}_{\eta }={L}_{\eta }\times \Delta {t}_{0}$	$40$
Gradient energy coeff.$\kappa$	$5\times {10}^{-5}\mathrm{J}/{\mathrm{m}}^{2}$	$\widetilde{\kappa }=\kappa /\left({E}_{0}\times {{l}_{0}}^{2}\right)$	$1\times {10}^{-2}$
Diffusion coeff. in electrode ${D}^{e}$	$7.19\times {10}^{-13}{\mathrm{m}}^{2}/\mathrm{s}$	${\widetilde{D}}^{e}={D}^{e}/\left({{l}_{0}}^{2}/\Delta {t}_{0}\right)$	$2.157\times {10}^{2}$
Diffusion coeff. in solution ${D}^{s}$	$7.19\times {10}^{-10}{\mathrm{m}}^{2}/\mathrm{s}$	${\widetilde{D}}^{s}={D}^{s}/\left({{l}_{0}}^{2}/\Delta {t}_{0}\right)$	$2.157\times {10}^{5}$
Time step $\Delta t$	$1.5\times {10}^{5}\mathrm{s}$	$\Delta \widetilde{t}=\Delta t/\Delta {t}_{0}$	$5\times {10}^{-3}$

where the characteristic length ${l}_{0}=100\mu m$, the characteristic time $\Delta {t}_{0}=3\times {10}^{9}s$, and the characteristic energy density ${E}_{0}=1.5\times {10}^{6}\mathrm{J}/{\mathrm{m}}^{3}$.

### Gaussian process regression

A Gaussian process is formed by assuming a zero-mean multivariant Gaussian prior distribution^36^

26 $$f\left({x}_{i}\right) \sim \mathcal{N}\left(f|0, K\right),$$

where $K$ is the covariance matrix, the element at $i$-th row $j$ th column is ${K}_{ij}=k({x}_{i},{x}_{j})$, $k$ is a covariance function that describes the relation between different inputs, zero mean is assumed for the prior.

Assuming the noise to be Gaussian with a variance of ${\sigma }_{y}^{2}$, the joint distribution between training data and test data is given by^36^

27 $$\left(\begin{array}{c}Y\\ {y}^{*}\end{array}\right)\sim \mathcal{N}\left(\begin{array}{c}Y\\ {y}^{*}\end{array}\bigg|0,\left[\begin{array}{cc}K\left(X,X\right)+{\sigma }_{y}^{2}\mathbf{I}& K(X, {x}^{*})\\ K({x}^{*}, X)& K({x}^{*},{x}^{*}) \end{array}\right]\right),$$

where ${x}^{*}$ represents the new input vector for the test data and ${y}^{*}$ represents corresponding predicted output, $K\left(X,X\right)$ is the covariance matrix between different training inputs with element $k({x}_{i},{x}_{j})$, $K({x}^{*}, X)$ denotes the covariance matrix between training inputs and new test inputs with element $K({x}^{*}, {x}_{j})$ and $K({x}^{*},{x}^{*})$ denotes the covariance between new test inputs.

Next, the Bayesian inference is used to calculate the posterior distribution over ${y}^{*}$^36^

28 $${y}^{*}\sim \mathcal{N}\left({y}^{*}|{\mu }^{*}\left({x}^{*}\right),{K}^{*}({x}^{*},{x}^{*}) \right),$$

where the mean of posterior prediction is ${\mu }^{*}\left({x}^{*}\right)=K({x}^{*}, X){\left[K({x}^{*},{x}^{*})+{\sigma }_{y}^{2}\mathbf{I}\right]}^{-1}K(X, {x}^{*})$, and the variance of posterior prediction is ${K}^{*}\left({x}^{*},{x}^{*}\right)=K\left({x}^{*},{x}^{*}\right)-K\left({x}^{*}, X\right){\left[K\left({x}^{*},{x}^{*}\right)+{\sigma }_{y}^{2}\mathbf{I}\right]}^{-1}K\left(X, {x}^{*}\right).$
